# Yeast Double Transporter Gene Deletion Library for Identification of Xenobiotic Carriers in Low or High Throughput

**DOI:** 10.1128/mbio.03221-21

**Published:** 2021-12-14

**Authors:** Ludimila Dias Almeida, Ali Salim Faraj Silva, Daniel Calixto Mota, Adrielle Ayumi Vasconcelos, Antônio Pedro Camargo, Gabriel Silva Pires, Monique Furlan, Helena Martins Ribeiro da Cunha Freire, Angélica Hollunder Klippel, Suélen Fernandes Silva, Cleslei Fernando Zanelli, Marcelo Falsarella Carazzolle, Stephen G. Oliver, Elizabeth Bilsland

**Affiliations:** a Synthetic Biology Laboratory, Department of Structural and Functional Biology, Institute of Biology, University of Campinas—UNICAMP, Campinas, São Paulo, Brazil; b Laboratory of Genomics and BioEnergy, Department of Genetics, Evolution, Microbiology and Immunology, Institute of Biology, University of Campinas—UNICAMP, Campinas, São Paulo, Brazil; c School of Pharmaceutical Sciences, São Paulo State University—UNESP, Araraquara, São Paulo, Brazil; d Chemistry Institute, São Paulo State University—UNESP, Araraquara, São Paulo, Brazil; e Cambridge Systems Biology Centre, University of Cambridge, Cambridge, United Kingdom; f Department of Biochemistry, University of Cambridge, Cambridge, United Kingdom; Tel Aviv University

**Keywords:** nonessential transporter double-deletion library, plasma membrane transporter, drug uptake, drug efflux, xenobiotics, *Saccharomyces cerevisiae*, drug transport, genetic interactions, yeast

## Abstract

The routes of uptake and efflux should be considered when developing new drugs so that they can effectively address their intracellular targets. As a general rule, drugs appear to enter cells via protein carriers that normally carry nutrients or metabolites. A previously developed pipeline that searched for drug transporters using Saccharomyces cerevisiae mutants carrying single-gene deletions identified import routes for most compounds tested. However, due to the redundancy of transporter functions, we propose that this methodology can be improved by utilizing double mutant strains in both low- and high-throughput screens. We constructed a library of over 14,000 strains harboring double deletions of genes encoding 122 nonessential plasma membrane transporters and performed low- and high-throughput screens identifying possible drug import routes for 23 compounds. In addition, the high-throughput assay enabled the identification of putative efflux routes for 21 compounds. Focusing on azole antifungals, we were able to identify the involvement of the *myo-*inositol transporter, Itr1p, in the uptake of these molecules and to confirm the role of Pdr5p in their export.

## INTRODUCTION

Novel drug candidates are generally designed based on the assumption that they enter cells by passive diffusion through the plasma membrane lipid bilayer. Thus, compounds that do not follow the rules predicting an efficient diffusion through the lipid bilayer are not considered drug-like and are discarded early in the drug discovery process. However, a growing body of evidence indicates that passive diffusion via the lipid bilayer is an exceptional, rather than the normal, mode of drug entry (1–6), with most drugs (and other xenobiotics) entering cells via protein carriers that normally carry nutrients or metabolites.

The investigation of the carrier substrate specificity is one of the objectives of the RESOLUTE consortium, a public-private partnership that aims to study the therapeutic potential of the human solute carrier (SLC) protein superfamily (7). This consortium works to create tools for studying these proteins on a large scale to associate specific classes of compounds with particular carriers. Therefore, knockout and tagged overexpression cell libraries are being built for most SLCs to carry out the “guilt-by-association” strategy. By using these approaches, the RESOLUTE consortium seeks to contribute to the inclusion of this superfamily of carriers in the class of classic drug targets.

**FIG 1:**
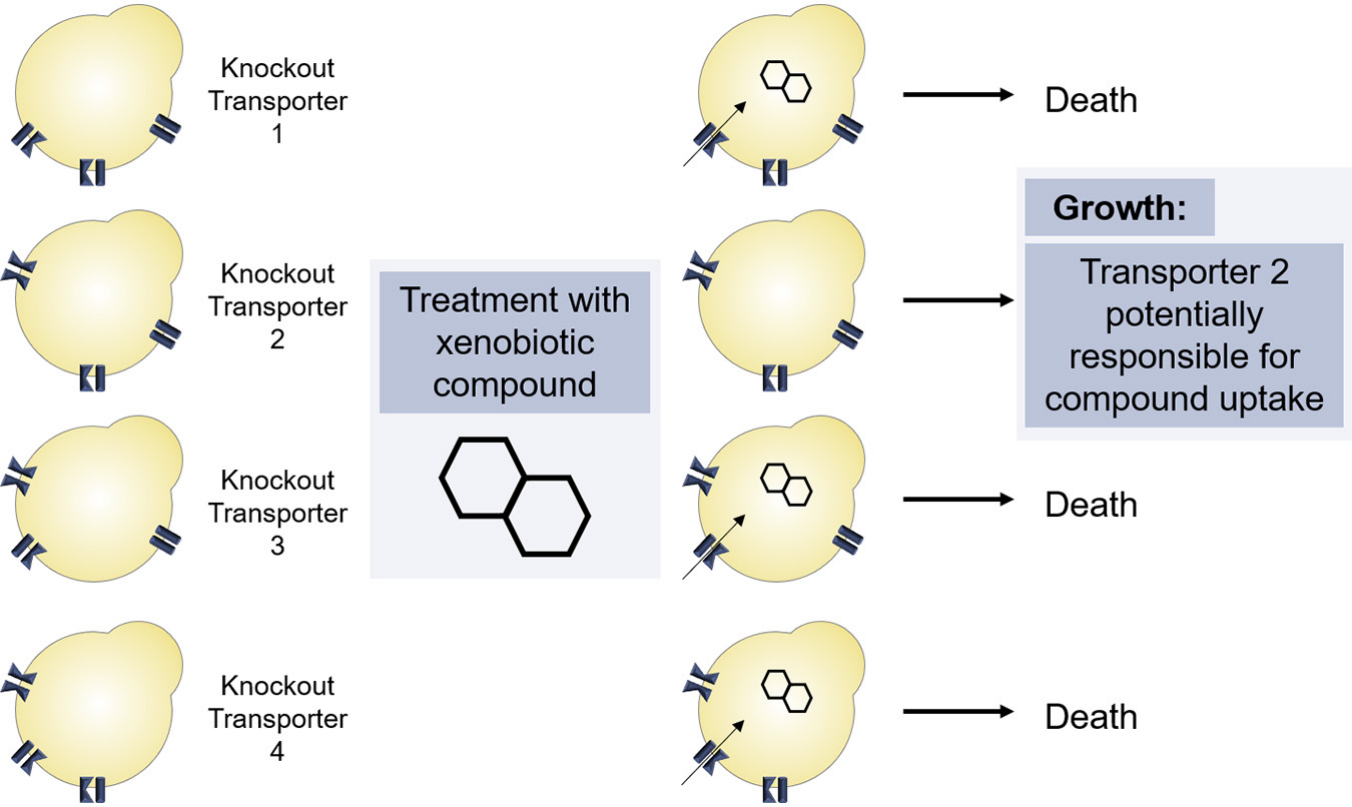
Schematic view of the experimental strategy designed to identify plasma membrane import routes. The strategy is applied for a cytotoxic compound with an intracellular target. For identification of a putative transporter, the deletion library of nonessential transporters is treated with inhibitory concentrations of the compound, and resistant strains are selected. These resistant strains probably lack the transporter responsible for the uptake of the tested compound. As exemplified, the deletion of transporter 2 interfered in the uptake of the xenobiotic and resulted in growth of this strain even in the presence of toxic concentrations of the tested molecule. As presented, transporter 2 is potentially responsible for the compound’s uptake.

Given the importance of mapping drug-transporter interactions to enabling a rational targeting of drugs to the tissues of interest, a method was developed to screen for yeast transmembrane proteins that mediated drug absorption; this identified the import routes for half of the screened anticancer compounds (8). The strategy assumes that a drug is toxic when present inside the cell in high concentration; thus, if the yeast does not have the carrier protein responsible for the entry of that molecule, it becomes drug resistant and survives (Fig. 1). Strains with deletions of individual genes encoding each of the nonessential transporters of the Saccharomyces cerevisiae plasma membrane were employed for transporter identification (8). The same approach was also used to study the specificity of human solute carriers on the import of 60 cytotoxic compounds using an SLC-specific CRISPR-Cas9 knockout (KO) library, suggesting the association between SLCs and the transport of 47 out of 60 compounds (∼80%) (9), a proportion similar to that observed in the yeast study (18/26; ∼70%) (8). In addition, CRISPR-Cas9 was also employed for large-scale transporter disruption in S. cerevisiae strains, aiming at the identification of carrier-mediated routes (10).

Yeast deletion libraries have been widely used, in genomic chemical profiling strategies, to study cellular responses to both new molecules and established drugs (11). In this approach, libraries with heterozygous deletions of all genes (haploinsufficiency profiling [HIP]) or homozygous nonessential genes (homozygous deletion profiling [HOP]) are employed in genetic screens to evaluate the cellular response to these molecules. Extensive studies have tested large numbers of molecules (12, 13) using these approaches, and the data are available to the research community. Based on the strategy previously presented for the identification of xenobiotic transporters, HOP data for strains with deletions of membrane transporters can provide insights into possible routes of entry for the screened molecules. However, the experimental design employed in most HOP assays aims to identify strains sensitive to low concentrations of test compounds and is not directly comparable to screens utilizing toxic concentrations (as required for transporter identification assays). Furthermore, proteins and drugs are highly promiscuous, with many drugs interacting with multiple off-target proteins in most cells (14). This promiscuity is not exclusive to the intracellular targets of drugs but also occurs in the transmembrane import and export of drug compounds (15).

**FIG 2:**
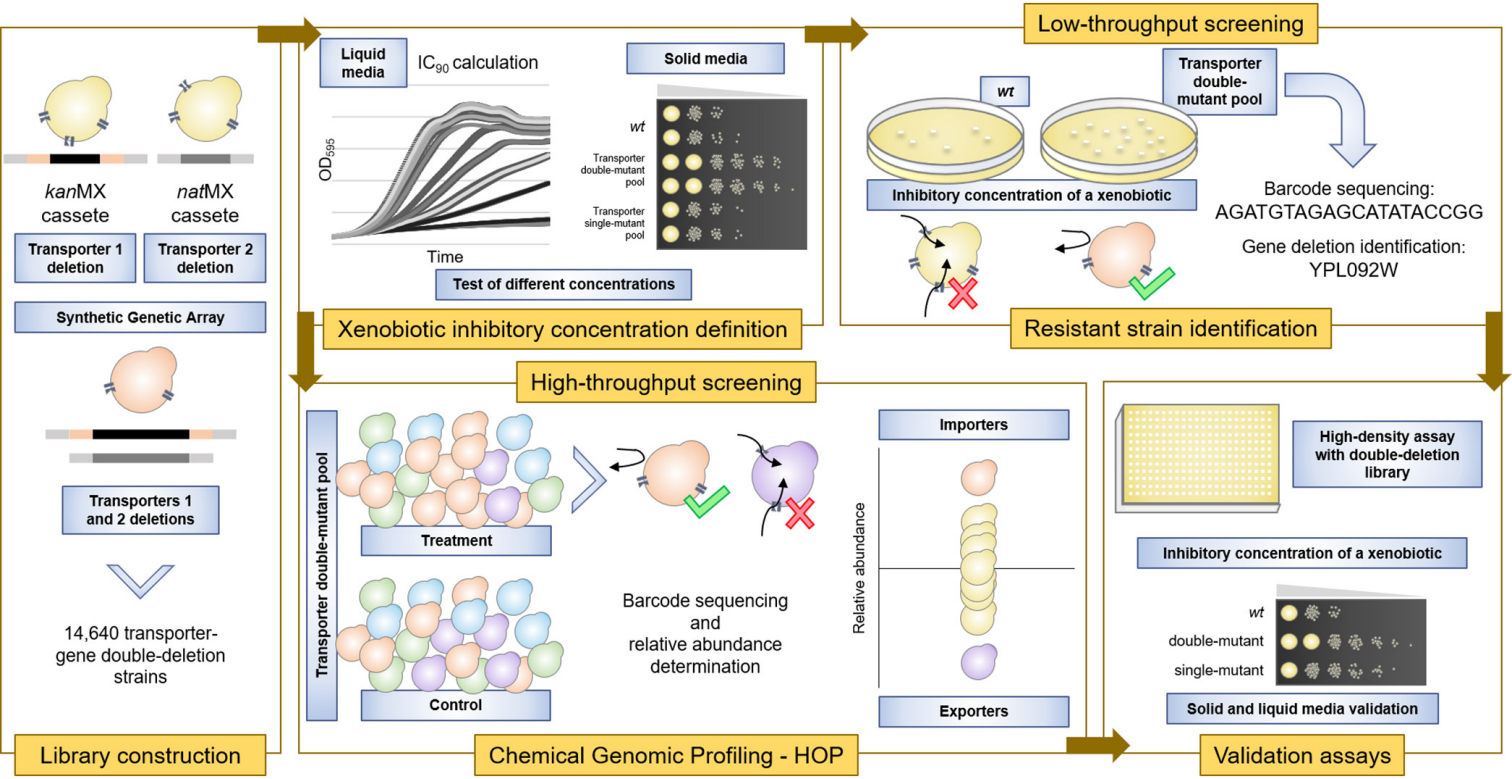
Experimental pipeline. Using the synthetic genetic array methodology, a library of double deletants of nonessential plasma membrane transporter-encoding genes was constructed to allow the rapid identification of *kan*MX deletions by barcode sequencing. Once the sublethal doses of commercial xenobiotics were defined, two approaches were employed for import route identification. A low-throughput screening in a plate-based assay was performed for selection of strains resistant to compounds, followed by barcode sequencing for identification of the transporter gene deletion responsible for the resistance phenotype. Chemical genomic profiling is a high-throughput approach employed to determine the relative abundance of transporter gene deletion strains in the presence of a xenobiotic, and thus suggest putative importers and exporters. In both strategies, validation assays were conducted for confirmation of putative transport routes. *wt*, wild type.

Due to the redundancy of transport functions between transmembrane proteins, we concluded that a larger set of drug import routes could be identified by testing drug import activity in strains lacking pairs of transporters. Hence, to screen for epistatic interactions between genes encoding transporter proteins, we constructed a library of S. cerevisiae strains containing double-deletion mutations of all pairwise combinations of genes that specify nonessential transmembrane transporters. We have characterized the performance of this double mutant collection in drug screening, employing both low- and high-throughput strategies (Fig. 2). These studies have reinforced our initial hypothesis that most drugs enter cells preferentially through plasma membrane transporters.

## RESULTS

### Double-deletion library construction.

The library with 14,640 strains carrying double deletions of genes encoding nonessential membrane transporters was built utilizing the synthetic genetic array (SGA) methodology. Genetic crosses were performed between a strain carrying a deletion due to replacement of a transporter gene with the *kan*MX cassette flanked by up- and downstream barcodes for tracking deletions and a second transporter deletant containing the *nat*MX replacement cassette but without the barcodes. Nonessential gene deletants were selected (from the library of strains available in the laboratory) based on their transporter function and plasma membrane expression (16).

### Commercial xenobiotics cytotoxic to yeast.

To allow a further characterization of our transporter double-deletion library and determine plasma membrane import routes for different xenobiotic compounds, we purchased 32 compounds that largely obey the Lipinski’s “rule of 5” (17) and are therefore expected to be preferentially imported into the cells by passive diffusion through the lipid bilayer (see Table S1 in the supplemental material). The carrier-mediated import route of 13 of these compounds had been evaluated previously (8), using a single-transporter gene deletion library. However, this earlier study was not able to define the specific transporter for all the tested compounds, perhaps due to carrier promiscuity.

10.1128/mbio.03221-21.1 TABLE S1 Xenobiotics used in this study. Compound name, catalogue numbers, and chemical properties. Download Table S1, PDF file, 0.1 MB. Copyright © 2021 Almeida et al. 2021 Almeida et al. https://creativecommons.org/licenses/by/4.0/ This content is distributed under the terms of the Creative Commons Attribution 4.0 International license.

**FIG 3:**
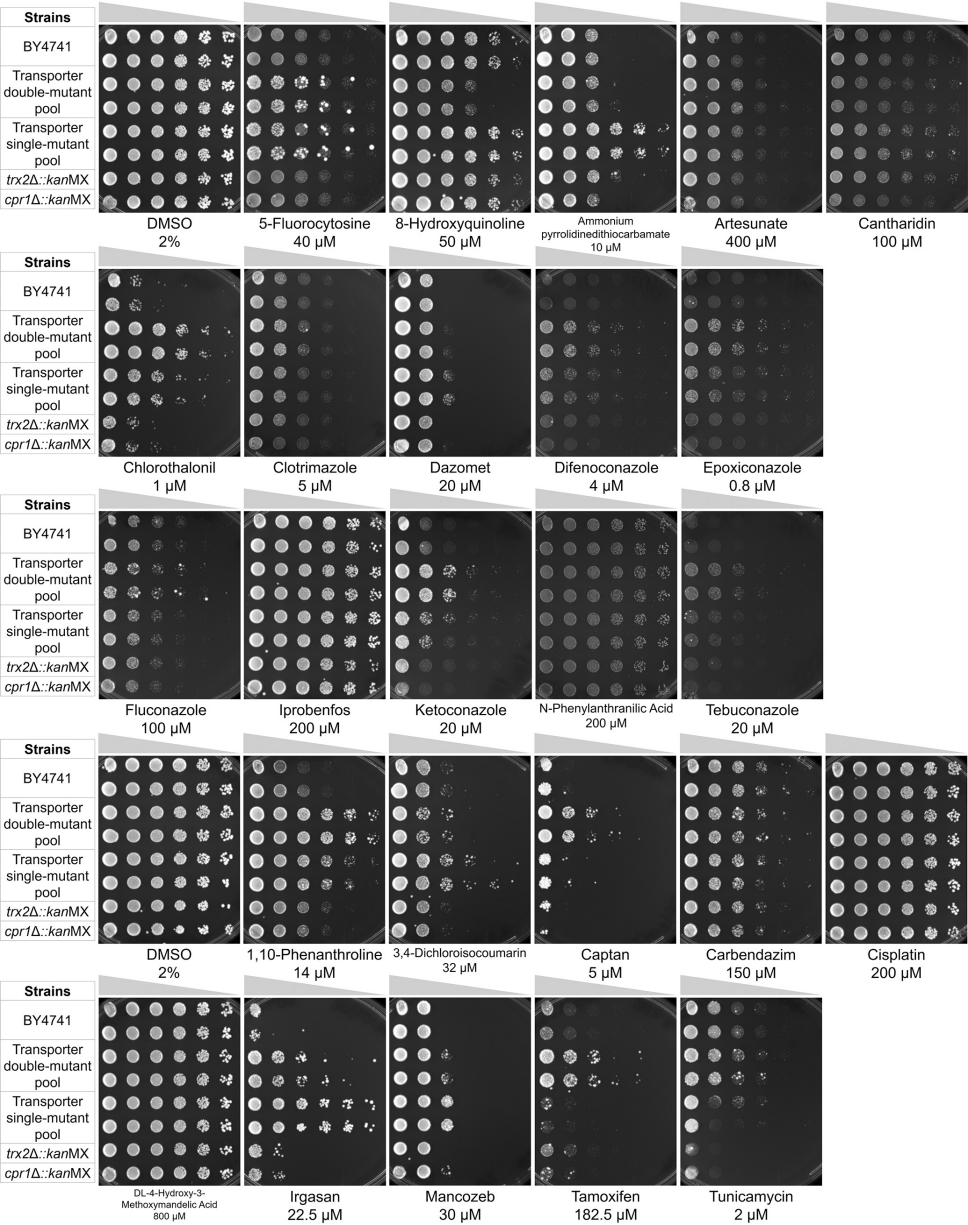
Spot test for inhibitory concentration definition. Serial dilutions (5×) of transporter gene double mutant and single mutant pool, wild-type (BY4741) and isolated single mutant (*trx2*Δ::*kan*MX and *cpr1*Δ::*kan*MX) S. cerevisiae strains spotted onto YNB+Sc plates containing the indicated concentrations of xenobiotics. For chlorothalonil, difenoconazole, epoxiconazole, fluconazole, ketoconazole, tebuconazole, 1,10-phenanthroline, captan, tamoxifen, and tunicamycin, it is possible to see that the double mutant pool library presents more resistant strains than the single mutant pool. For ammonium pyrrolidinedithiocarbamate, 3,4-dichloroisocoumarin, irgasan, and mancozeb, the single mutant library presents more resistant strains, which may be due to the group of deletions not represented in the double gene deletion library or because of the cell background. 5-Fluorocytosine shows a very similar pattern in the two libraries, and 8-hydroxyquinoline shows a pattern that seems to correspond to a cell background from the double deletion library. Other compounds did not present a selective cytotoxicity between libraries.

We determined an approximate 90% inhibitory concentration (IC_90_) for each compound’s effect on yeast in liquid cultures and proceeded to select for resistance in plates with sublethal doses of each compound. We performed serial dilutions of the wild type (BY4741), the transporter double mutant, and single-mutant pools and spotted these onto YNB+Sc (6.7 g/liter yeast nitrogen base with ammonium sulfate and without amino acids, complete amino acid supplement, and 2% glucose) agar plates with inhibitory concentrations of the commercial xenobiotics (Fig. 3). Strains with single-gene deletion mutations in genes encoding cytoplasmic nontransporter proteins were included (*trx2*Δ::*kan*MX and *cpr1*Δ::*kan*MX) as controls. The xenobiotics for which we did not observe significant growth inhibition at 200 μM (maximum of 2% dimethyl sulfoxide [DMSO]; 400 μM for artesunate and 800 μM for dl-4-hydroxy-3-methoxymandelic acid and tamoxifen) in solid media were excluded from further screens (Fig. 3).

Using spot assays, we defined which compounds were toxic to yeast in solid media and the appropriate concentration to inhibit the growth of the wild-type strain while selecting for resistant strains from the library pool. For some compounds, we were able to observe a clear difference in resistance between the wild type and the transporter deletants, as shown in Fig. 3. In addition, we were able to identify compound-inhibitory differences between double- and single-deletant libraries. It is expected, for a compound imported by a transporter, that the double-deletion library will present double the number of resistant strains than the single-deletion library, even when only one transporter is involved in the uptake. We saw this pattern on using inhibitory concentrations of 1,2,4-triazoles (epoxiconazole, difenoconazole, and tebuconazole) and chlorothalonil, for example (Fig. 3). Thus, working with a double-deletion library can facilitate the identification of transporters. However, in several cases, we observed more colonies in the single-deletion library that could be due to differences in strain background, as a number of different markers were introduced in the double mutant strains to allow large-scale selection of the desired haploids.

We employed two approaches for compound transporter identification: a low- and a high-throughput strategy. The low-throughout method is a plate-based screen with the selection of resistant strains in the presence of inhibitory concentrations of xenobiotics and identification of transporter deletions bearing the *kan*MX cassette by barcode sequencing. This strategy allows a visual assessment of a possible involvement of transporters in drug uptake and the identification of candidate import routes. The second approach was the high-throughput screening, where we evaluated the fluctuation of abundance of transporter deletion strains by sequencing the *kan*MX upstream barcodes from the library pool in a liquid culture containing inhibitory concentrations of a compound. This screening allowed us to identify not only strains resistant to a given compound but also strains sensitive to this compound.

### Low-throughput (plate-based) strategy for xenobiotic transporter identification.

**FIG 4:**
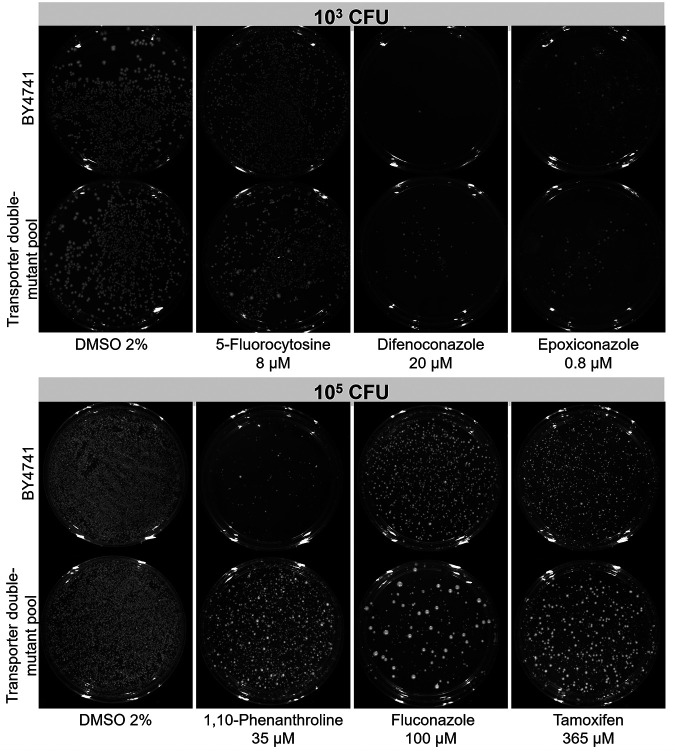
Selection of transporter gene deletion strains resistant to xenobiotics. Approximately 10^3^ or 10^5^ CFU of wild type (BY4741) or transporter gene deletion pool were plated onto YNB+Sc agar plates with the indicated concentrations of xenobiotics (or solvent control [DMSO 2%]) to identify putative differences in the number and size of drug-resistant colonies. Resistant transporter gene deletion colonies were picked for barcode identification.

Xenobiotic-resistant strains from the transporter double-deletion library pool were selected by plating 10^6^, 10^5^, 10^4^, and 10^3^ CFU of wild-type BY4741 or the transporter deletion pool onto YNB+Sc agar plates containing inhibitory concentrations of the compounds (Fig. 4; data not shown). We expected to observe an even growth inhibition of the wild-type strain and the appearance of resistant colonies (corresponding to the deletion of genes encoding transporters responsible for drug uptake) on plates where the compounds enter cells through plasma membrane transporters. For the selection of xenobiotic-resistant strains from the library pool, we investigated the difference between the number of resistant colonies, aiming to define conditions in which more deletant colonies could grow on the pool plate in comparison to that of the wild type. However, we also observed cases in which the greatest differences between the wild type and transporter deletion pools were the formation of colonies with different sizes; hence, we collected the largest colonies to identify the deleted carrier gene. This was the case for 5-fluorocytosine and fluconazole, for example (Fig. 4). Once 20 to 40 resistant colonies were selected for each xenobiotic, barcodes (associated with the *kan*MX cassette) from approximately 20 strains were sequenced to identify the transporter genes that had been deleted.

After aligning the sequenced barcodes to the *Saccharomyces* Genome Deletion Project’s barcode list (http://www-sequence.stanford.edu/group/yeast_deletion_project/deletions3.html), we identified the genes deleted in each compound-resistant strain (Table 1). It is important to note that, as only the genes deleted with *kan*MX were barcoded, the sequencing results show only one of the transporter-encoding genes deleted in the compound-resistant strain. Furthermore, as during the library construction, we had several copies of the *his3* deletion in all mating plates, this deletion is present in high frequency in the library. Hence, *HIS3* “hits” were excluded from further data analysis. As expected, for 5-fluorocytosine, the positive control of the study, 7 out of 10 of the carriers identified by deletion with the *kan*MX cassette correspond to Fcy2p (gene *FCY2* [YER056C]). For the other three 5-fluorocytosine-resistant strains selected, the deletion of *FCY2* was identified by PCR with the *nat*MX cassette (data not shown).

**TABLE 1:** Transporter gene deletion strains resistant to xenobiotics in agar plates (low throughput) or liquid cultures (high throughput)

Compound	Low-throughput assay		High-throughput assay	
	Concn (μM)	Hits^*a*^	Concn (μM)	Hits^*b*^ (log_2_ fold change)
5-Fluorocytosine	8	**7× *fcy2*Δ,** *ftr1*Δ*, tat2*Δ	20	*fui1*Δ (2.22), *tat1*Δ (1.23), *adp1*Δ (1.27), *bap3*Δ (0.93), ***fcy2*Δ (8.44),** *azr1*Δ (1.06), *gal2*Δ (0.59), *atr2*Δ (0.96), *nrt1*Δ (1.24), *pdr12*Δ (1.84), *ssu1*Δ (2.68), *aqy1*Δ (1.14)
Clotrimazole	10	*fui1*Δ*, mal31*Δ*, tpo2*Δ*, cch1*Δ*, mal11*Δ*, arn2*Δ, 2× *hxt1*Δ*, opt1*Δ, **4× *nha1*Δ***, mep2*Δ*, lyp1*Δ*, alp1*Δ*, thi72*Δ*, pma2*Δ	2	***nha1*Δ (0.72)**
Ketoconazole	25	*gnp1*Δ*, mep1*Δ*, tok1*Δ*, jen1*Δ, **14× *nha1*Δ**	10	*adp1*Δ (0.56), ***nha1***Δ **(1.10)**
Difenoconazole pestanal	20	*dtr1*Δ*, snq2*Δ, ***itr1*Δ***, gnp1*Δ, ***tna1*Δ***, arn1*Δ*, arn2*Δ*, gap1*Δ, **6× *nha1*Δ***, mmt1*Δ	0.16	*adp1*Δ (0.70), ***itr1*Δ (0.90)**, *ftr1*Δ (1.08), ***tna1*Δ (0.63)**, *yor1*Δ (0.81), *tpo5*Δ (1.35), *tpo1*Δ (0.91), ***nha1*Δ (2.51)**, *smf1*Δ (0.93), *tpo4*Δ (0.73)
Epoxiconazole pestanal	0.8	*pho89*Δ, 2× *hxt3*Δ*, ato3*Δ, **2× *itr1*Δ**, 2× *cch1*Δ*, dur3*Δ*, hxt5*Δ*, nft1*Δ, ***ybt1*Δ***, mmt1*Δ*, mch5*Δ*, pma2*Δ*, aqy1*Δ	0.032	*adp1*Δ (0.75), *snq2*Δ (1.12), ***itr1*Δ (1.52),** *ftr1*Δ (1.94), *tna1*Δ (0.67), *yor1*Δ (0.97), *gef1*Δ (0.69), *tpo5*Δ (1.96), *tpo1*Δ (1.11), ***ybt1*Δ (0.61),** *nha1*Δ (3.93), *smf1*Δ (1.51), *tpo4*Δ (0.76), *dip5*Δ (0.67), *ctr1*Δ (0.76)
Tebuconazole pestanal	20	*bap3*Δ, **2× *itr1*Δ***, agp3*Δ*, hnm1*Δ, 2× *mep1*Δ*, hxt8*Δ*, nft1*Δ*, prm6*Δ*, atr1*Δ*, fet4*Δ, ***tpo4*Δ***, mch5*Δ*, pma2*Δ*, aqy1*Δ	0.32	*adp1*Δ (0.69), ***itr1*Δ (1.04)**, *ftr1*Δ (0.94), *tna1*Δ (0.82), *yor1*Δ (0.81), *tpo5*Δ (1.19), *tpo1*Δ (0.73), *nha1*Δ (2.67), *smf1*Δ (0.84), ***tpo4*Δ (0.53)**, *ctr1*Δ (0.65)
Fluconazole	100	**3× *qdr3*Δ*, tat1*Δ***, agp1*Δ, ***ady2*Δ*, adp1*Δ**, ***yor1*Δ*, arn1*Δ*, hxt4*Δ,** *gex2*Δ***, bor1*Δ*, hol1*Δ***, enb1*Δ*, nrt1*Δ*, ssu1*Δ*, aqy1*Δ	75	*seo1*Δ (1.60), *flr1*Δ (1.83), ***qdr3*Δ (2.72),** *bap2*Δ (2.57), ***tat1*Δ (2.66),** *pca1*Δ (1.76), *pho89*Δ (1.50), *mal31*Δ (2.57), ***ady2*Δ (1.73), *adp1*Δ (2.48),** *git1*Δ (1.66), *sit1*Δ (1.62), ***yor1*Δ (2.54), *arn1*Δ (1.98), *hxt4*Δ (2.79),** *hxt1*Δ (2.68), *hxt5*Δ (2.39), *qdr2*Δ (5.96), ***bor1*Δ (1.17), *hol1*Δ (5.57),** *mch5*Δ (1.54)
1,10-Phenanthroline	35	2× *fui1*Δ*, adp1*Δ*, itr1*Δ*, fcy2*Δ*, tpo2*Δ*, arn1*Δ*, arn2*Δ*, tok1*Δ*, mid1*Δ*, enb1*Δ*, pdr5*Δ	10	No hits
3,4-Dichloroisocoumarin	16	*itr1*Δ, YFL040WΔ*, hxt1*Δ*, kch1*Δ*, mmp1*Δ*, prm6*Δ*, lyp1*Δ, 2× *thi72*Δ, 2× *ssu1*Δ*, dip5*Δ	10	No hits
8-Hydroxyquinoline	7, 25, 50	*pca1*Δ, 2× *fen2*Δ*, sit1*Δ*, ftr1*Δ*, mep1*Δ, 2× *arn2*Δ*, qdr1*Δ*, 2× dal5*Δ*, hol1*Δ*, aus1*Δ	50	*ctr1*Δ (1.35)
Ammonium pyrrolidinedithiocarbamate	4	*dtr1*Δ*, zrt1*Δ*, pdr11*Δ*, itr2*Δ	NP	NP
Artesunate	400	2× *flr1*Δ, 2× *can1*Δ, YFL040WΔ*, alr2*Δ*, agp3*Δ*, arn1*Δ*, hxt4*Δ, ***tpo1*Δ,** *ybt1*Δ*, atr2*Δ*, bor1*Δ	200	*tna1*Δ (0.79), ***tpo1*Δ (0.72),** *nha1*Δ (0.91), *hxt17*Δ (0.72)
Cantharidin	60	2× *pca1*Δ*, fen2*Δ*, zrt1*Δ*, yor1*Δ*, dur3*Δ, 2× *hxt1*Δ, *pdr11*Δ*, qdr1*Δ*, trk1*Δ*, hxt14*Δ*, itr2*Δ*, tpo4*Δ	NP	NP
Captan pestanal	5	*dtr1*Δ*, bap3*Δ*, fcy2*Δ*, kch1*Δ*, jen1*Δ*, prm6*Δ*, mid1*Δ*, tpo4*Δ*, mch5*Δ*, put4*Δ*, sam3*Δ*, sge1*Δ	1.6	No hits
Carbendazim	150, 200	*agp1*Δ*, ady2*Δ*, adp1*Δ*, ato3*Δ, ***tna1*Δ***, yor1*Δ*, pdr11*Δ*, kch1*Δ***, nft1*Δ***, ybt1*Δ, ***nha1*Δ***, atr1*Δ*, hxt14*Δ, ***pdr5*Δ**	200	*fur4*Δ (0.77), *tat1*Δ (1.77), *cin10*Δ (0.99), *hnm1*Δ (0.55), ***tna1*Δ (2.01), *nft1*Δ (0.77), *nha1*Δ (2.04),** *aqr1*Δ (0.53), *tat2*Δ (1.08), ***pdr5*Δ (1.29),** *ctr1*Δ (0.99)
Chlorothalonil pestanal	0.1, 1	*fur4*Δ*, hxt3*Δ*, ato3*Δ*, gnp1*Δ, 4× *zrt1*Δ*, nft1*Δ*, sul2*Δ, ***atr1*Δ***, bor1*Δ*, bio5*Δ*, pdr5*Δ*, mch5*Δ*, dip5*Δ	0.8	***atr1*Δ (0.77)**
Dazomet pestanal	15, 20	3× YDL199CΔ*, gex2*Δ*, mep2*Δ*, smf1*Δ*, ssu1*Δ*, tpo3*Δ	NP	NP
Iprobenfos pestanal	200	*flr1*Δ*, qdr3*Δ*, git1*Δ*, hxt10*Δ*, mal11*Δ*, sul2*Δ*, hxt2*Δ*, aus1*Δ*, thi72*Δ*, tpo4*Δ*, put4*Δ*, ssu1*Δ*, aqy1*Δ	200	No hits
Irgasan	40, 50	*pca1*Δ*, stl1*Δ*, alr2*Δ*, trk1*Δ*, aqr1*Δ, 2× *mep2*Δ*, lyp1*Δ, 2× *mid1*Δ*, ato2*Δ, 2× *aus1*Δ*, sam3*Δ	15	*fen2*Δ (1.94), *arn1*Δ (0.52), *arn2*Δ (0.69)
Mancozeb pestanal	10, 20, 30	*sul1*Δ*, stl1*Δ*, zrt1*Δ*, opt1*Δ*, nha1*Δ*, atr1*Δ	NP	NP
*N*-Phenylanthranilic acid		No resistance selected	100	*snq2*Δ (0.68), *tna1*Δ (0.87), *mid1*Δ (0.76), *pdr5*Δ (1.01)
Tamoxifen	365	YDL199CΔ*, opt1*Δ*, kch1*Δ*, dal5*Δ, 7× *tpo5*Δ, 3× *nha1*Δ*, atr1*Δ*, mep2*Δ*, itr2*Δ*, mch5*Δ	40	*adp1*Δ (0.50), *tna1*Δ (1.18)
Tunicamycin	4	*fui1*Δ*, flr1*Δ, 11× *fur4*Δ*, qdr3*Δ*, mal31*Δ*, zrt1*Δ*, nft1*Δ*, tpo4*Δ*, aqy1*Δ	1	No hits

a Bold deletant names correspond to those that are repeated between low- and high-throughput screenings.

b Log_2_ fold change ≥ 0.5; *P* value adjusted for multiple tests (padj) ≤ 0.1; *P* value ≤ 0.001. NP, not performed.

Analyzing the results in plate assays (Table 1), we could observe recurrent transporter gene deletions for some compounds. This is best exemplified by the ketoconazole results, where 14 resistant strains were *nha1*Δ strains. Other compounds showed preferential representation of certain transporter gene deletion strains, indicating their potential involvement in compound import. Although these results suggested possible transporters, additional assays are needed to confirm these phenotypes. In fact, plate screening assays can give rise to false-positive results, requiring a larger sample size to confirm the hits. Thus, for a more comprehensive screening, with the monitoring of the fluctuation in the abundance of all strains in the pool, we performed a high-throughput assay in liquid culture.

### High-throughput (liquid growth) strategy for xenobiotic transporter identification.

**FIG 5:**
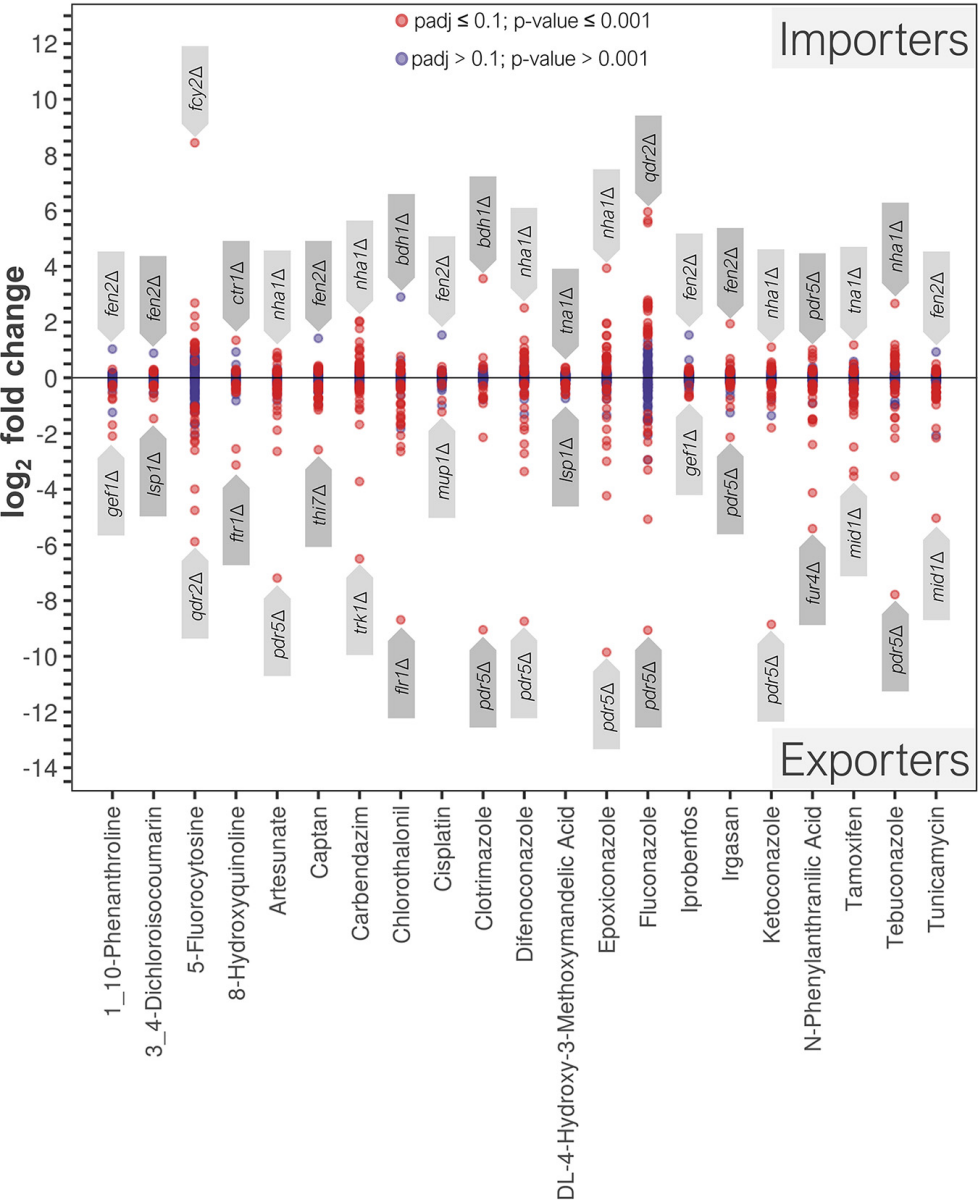
Relative abundance of double mutant library strains in the presence of cytotoxic concentrations of xenobiotics evaluated by CGP. Resistant strains (log_2_ fold change > 0) indicate the potential involvement of the protein encoded by the deleted gene in the uptake of the xenobiotic (Importers). Sensitive strains (log_2_ fold change < 0) indicate the potential involvement of the deleted gene’s product in efflux of the xenobiotic (Exporters). Strains with the highest and lowest abundance were labeled for identification.

The putative import and export routes of xenobiotic compounds were investigated by chemical genomic profiling (CGP) using our double mutant nonessential transporter gene deletion library. The library was cultivated in liquid cultures containing inhibitory concentrations of xenobiotics for ca. 15 generations, and the *kan*MX upstream barcodes from the population were sequenced for transporter gene deletion identification. Thus, we were able to identify deletions responsible for resistance (putative import route) and sensitivity (putative export route) to the test compounds (Fig. 5 and Table 1; also see Data Set S1 in the supplemental material). Treatment with 5-fluorocytosine identified the *fcy2*Δ mutant as the most abundant strain of the assay, confirming the accuracy of the method.

10.1128/mbio.03221-21.10 DATA SET S1 Differential abundance of barcodes between samples treated compared to untreated controls of 21 compounds. Each sheet corresponds to data from each compound, specified in the sheet name. Headlines are described on the first sheet (headline meaning). Download Data Set S1, XLSX file, 0.3 MB. Copyright © 2021 Almeida et al. 2021 Almeida et al. https://creativecommons.org/licenses/by/4.0/ This content is distributed under the terms of the Creative Commons Attribution 4.0 International license.

Among the significantly less abundant strains (*P* value adjusted for multiple tests [padj] of ≤0.1; *P* value ≤ 0.001; log_2_ fold change ≤ 0.5), we could identify deletions for genes encoding ATP-binding cassette (ABC) drug efflux pumps involved in pleiotropic drug resistance: Pdr5p, Snq2p, and Yor1p (18) (Data Set S1). The *pdr5*Δ mutation caused sensitivity to artesunate, irgasan, iprobenfos, and azoles, the latter being consistent with published results (19, 20). The *snq2*Δ mutation caused sensitivity to artesunate, carbendazim, and chlorothalonil. The *yor1*Δ mutation caused sensitivity only to tunicamycin. Pdr11p, a pleiotropic drug resistance (PDR) family member involved in sterol uptake (21, 63), was identified among mutations that conferred sensitivity to artesunate, captan, chlorothalonil, difenoconazole, *N*-phenylanthranilic acid, tamoxifen, and tunicamycin. The PDR11 paralog, AUS1, involved in sterol uptake (21), was not represented among depleted strains. We also identified Nft1p, a putative transporter of the multidrug resistance protein (MRP) subfamily (22, 63), as a candidate exporter for epoxiconazole, tebuconazole, difenoconazole, and 5-fluorocytosine.

Multidrug resistance transporters can also belong to the major facilitator superfamily (MFS) (23, 24); however, these had little impact on sensitivity to our test compounds, with the following exceptions: *flr1*Δ (chlorothalonil and 3,4-dicloroisocoumarin), *dtr1*Δ (fluconazole), *qdr1*Δ (5-fluorocytosine), *qdr2*Δ (5-fluorocytosine), and *atr1*Δ (5-fluorocytosine). It should be noted that some transporters of this class (e.g., Flr1p) have been reported to be determinants of resistance to compounds tested in this study (e.g., fluconazole) (25); however, our CGP did not confirm these correlations.

**FIG 6:**
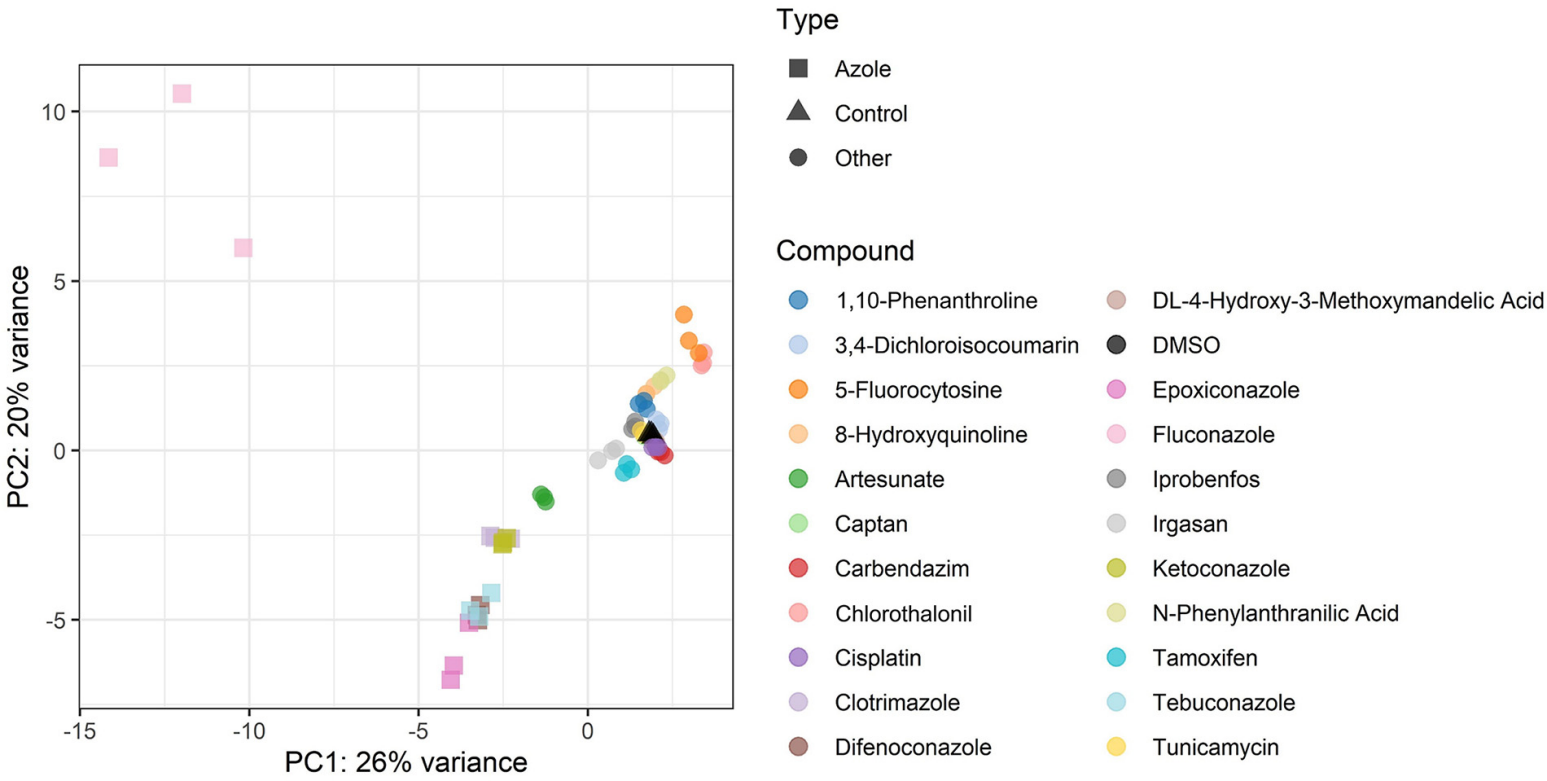
Principal-component analysis of all treatments from CGP performed with our transporter gene double deletion library and sublethal dose of xenobiotics. Azole compounds group in the PCA plot, with the exception of fluconazole. Agrochemical triazoles (difenoconazole, epoxiconazole, and tebuconazole) and imidazoles (clotrimazole and ketoconazole) form two subgroups. This may indicate a similar strain composition profile and probably an involvement of a set of transporter proteins in the carriage of these compounds across the cell membrane.

Principal-component analysis (PCA) allows an evaluation of the distribution of variations between replicates and conditions within an experiment. Figure 6 and Fig. S1 in the supplemental material show that the strain composition in the pool following some treatments is very similar to the control (DMSO) in both the first and second principal components. It is important to note that replicates of each xenobiotic cluster within the same region of the PCA plot and that the azoles tested (with the exception of fluconazole) present very similar strain composition profiles, which validates the reproducibility of the experiments. We would also note that 5-fluorocytosine (Fig. S1) shows a very different profile compared to all other treatments in the analysis, with the exception of fluconazole (Fig. 6).

10.1128/mbio.03221-21.2 FIG S1 Principal component analysis of all treatments from the CGP experiment, excluding fluconazole. Exclusion of fluconazole from PCA plot revealed a different profile for 5-fluorocytosine, the positive control of the assay. Azoles still group, with a clear separation of the subgroups triazoles and imidazoles. Download FIG S1, PDF file, 2.8 MB. Copyright © 2021 Almeida et al. 2021 Almeida et al. https://creativecommons.org/licenses/by/4.0/ This content is distributed under the terms of the Creative Commons Attribution 4.0 International license.

### Overview of putative import routes for the xenobiotics.

Some of the compounds evaluated in our assays were not cytotoxic in either liquid or solid medium at the highest concentrations tested (typically 200 μM compound corresponding to 2% DMSO in the medium) which prevented us from performing the downstream experiments to identify resistant strains and suggest import routes. To propose possible transport routes for all the other compounds tested, we evaluated the results obtained from both low- and high-throughput assays (Table 1). In addition, we performed high-density plate-based assays with 308 strains selected from the double-deletion library to evaluate the profile of resistance in the presence of the xenobiotics tested. We highlight below some interesting results and propose transporter relationships for some compounds.

Tunicamycin is a nucleoside antibiotic that inhibits N-glycosylation of asparagine in eukaryotes, and the use of this substance is important for the study of the UPR (unfolded protein response) signaling network (26). In the plate assay, we identified Fur4p as a possible tunicamycin carrier, since its deletion was present in 11 of the 20 resistant colonies analyzed (Table 1). Considering the CGP assay, however, the *fur4*Δ deletion strain did not appear significantly abundant (Table 1). However, in the high-density assay, the double mutant *pdr5*Δ::*nat*MX *fur4*Δ::*kan*MX showed a resistant phenotype (F18; Fig. S2). The reciprocal double mutant, *fur4*Δ::*nat*MX *pdr5*Δ::*kan*MX did not grow in either the treatment or control plates (I03; Fig. S2). It is possible to identify a plate effect in this row, in which more double mutants show resistance, and this result should be carefully evaluated. Fur4p acts as a uracil permease (27, 28), which indicates that its contribution to the entry of the compound tunicamycin may be due to interaction with the uracil moiety present in the structure of this compound. In a previous study (8), the transporters Lem3p, Dnf2p, and Qdr2p were identified as responsible for the entry of tunicamycin into the cell. The absence of Lem3 and Dnf2 transporters in the double mutant library tested in our work and of *fur4*Δ among the deletions tested previously (8) prevents the cross-validation of the two results. Thus, the previous and current results indicate the involvement of Dnf2p, Lem3p, and Fur4p in the uptake of tunicamycin.

10.1128/mbio.03221-21.3 FIG S2 High-density assay with 308 double-deletion strains (in quadruplicate) in the presence of an inhibitory concentration of tunicamycin. The upper left panel shows a test plate containing tunicamycin (4 days of growth). The upper right panel shows a control plate with 2% DMSO (4 days of growth). The middle panel presents a plate map (::*nat*MX followed by ::*kan*MX), and the lower panel shows the z-score for each quadruplicate. Green squares correspond to scores 3*SD, and orange squares correspond to between 2*SD and 3*SD. The high-density assay presents the same 2% DMSO plate as the experiment control. Download FIG S2, TIF file, 2.5 MB. Copyright © 2021 Almeida et al. 2021 Almeida et al. https://creativecommons.org/licenses/by/4.0/ This content is distributed under the terms of the Creative Commons Attribution 4.0 International license.

Tamoxifen is an antitumorigenic selective estrogen receptor modulator (29, 30). The plate assay and the high-density assay revealed that deletions of the *TPO5* and *NHA1* genes resulted in resistance to the compound in solid medium (Table 1; Fig. S3). Nha1p acts as a cation antiporter (31–33), and Tpo5p is a putrescine and spermidine exporter; however, it localizes to the Golgi and post-Golgi vesicles (34). In the high-density assay, however, only the *tpo5*Δ::*nat*MX *nha1*Δ::*kan*MX double mutant showed a resistance phenotype, and the *nha1*Δ::*nat*MX *tpo5*Δ::*kan*MX strain was not resistant (Fig. S3). It is important to note that the *nha1*Δ::*kan*MX deletion appears in other resistant strains obtained in the high-density assay, which may indicate that the resistance is due to a strain background effect and not a specific consequence of the transporter deletion. To investigate this possibility, we performed spot tests with *nha1*Δ::*kan*MX and *nha1*Δ::*nat*MX mutants in combination with 12 different transporters and *his3* strain as a negative control (data not shown). In all cases, we observed that *nha1*Δ::*kan*MX conferred resistance to tamoxifen, whereas the same was not always evident for *nha1*Δ::*nat*MX. *nha1*Δ::*nat*MX was resistant to tamoxifen in combination with approximately 50% of the transporter deletions tested: *itr1*Δ::*kan*MX, *tpo5*Δ::*kan*MX, *mal11*Δ::*kan*MX, *zrc1*Δ::*kan*MX, *dnf1*Δ::*kan*MX, or *fcy2*Δ::*kan*MX (none of which was previously identified as resistant to the drug). This suggests that there could have been a mutation in the original *nha1*Δ::*nat*MX that leads to an increased sensitivity to tamoxifen and is present in half of the spores produced in the library. The same pattern was not evident for azoles. Considering the CGP assay, the most abundant strains (log_2_ fold change ≥ 0.5) in the tamoxifen-treated pool were the *adp1*Δ and *tna1*Δ mutants (Table 1). Adp1 is a putative ATP-dependent permease (35), and Tna1p is a high-affinity nicotinic acid permease (36). Although the *tpo5*Δ and *nha1*Δ mutants had a log_2_ fold change above zero, it was not greater than 0.5. Thus, there is a clear difference in resistance between cells grown on solid media and in liquid media.

10.1128/mbio.03221-21.4 FIG S3 High-density assay with 308 double-deletion strains (in quadruplicate) in the presence of an inhibitory concentration of tamoxifen. The upper left panel shows a test plate containing tamoxifen (4 days of growth). The upper right panel shows a control plate with DMSO 2% (4 days of growth). The middle panel presents a plate map (::*nat*MX followed by ::*kan*MX), and the lower panel shows the z-score for each quadruplicate. Green squares correspond to scores 3*SD, and orange squares correspond to between 2 and 3*SD. The high-density assay presents the same 2% DMSO plate as the experiment control. Download FIG S3, TIF file, 2.5 MB. Copyright © 2021 Almeida et al. 2021 Almeida et al. https://creativecommons.org/licenses/by/4.0/ This content is distributed under the terms of the Creative Commons Attribution 4.0 International license.

Carbendazim is a benzimidazolic carbamate fungicide that acts by inhibiting the polymerization of microtubules by interaction with β-tubulin (37). Some carrier deletions were found as resistant strains in the plate assay and among the abundant strains of the CGP (Table 1). Considering those strains with a log_2_ fold change of  ≥0.5, we have the *tna1*Δ, *nft1*Δ, *nha1*Δ, and *pdr5*Δ strains; with a log_2_ fold change between 0 < 0.5, we have *yor1*Δ, *ady2*Δ, and *ybt1*Δ strains. Tna1p is a high-affinity transporter of nicotinic acid, a pyridinecarboxylic acid (36), and may be directly involved in the entry of carbendazim. It is worth noting the presence of the carboxylic acid group among the natural substrates for transporters identified as hits for carbendazim, as nicotinic acid for Tna1p and acetate for Ady2p. On the other hand, Nha1p may make only an indirect contribution to the drug’s ingress due to its function as a cation antiporter. It is noteworthy that we identified the ABC family members Yor1p, Ybt1p, and Nft1p (multidrug resistance protein [MRP] subfamily) (22, 38) as putative importers. We also identified Pdr5p as a putative importer of carbendazim, which is interesting since this protein is typically described as an exporter. Validation plate assays using the *pdr5*Δ single mutant growing in an inhibitory concentration of carbendazim (data not shown) indicated that this deletion provides resistance to this compound, corroborating the involvement of Pdr5p in carbendazim uptake.

**FIG 7:**
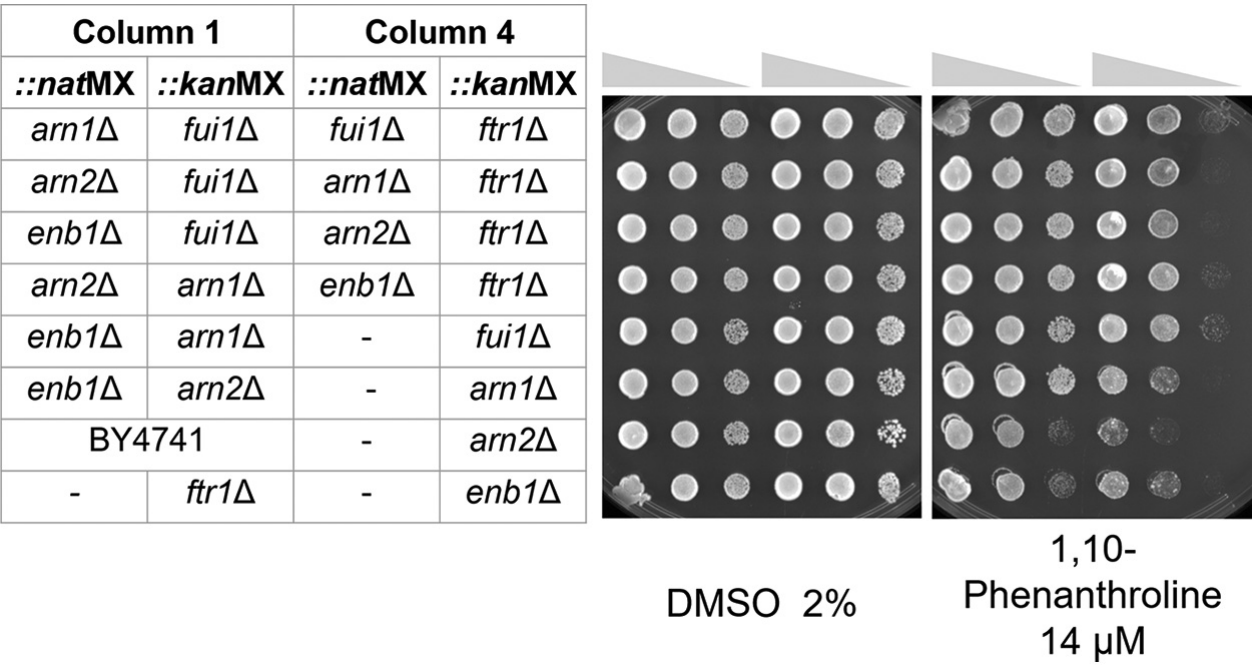
Validation of double mutant resistance to 1,10-phenanthroline. The panels present a spot test of serial dilutions of double-deletion strains in the presence of 1,10-phenanthroline or 2% DMSO control. Combinations of mutations in genes encoding large-molecule transporters (Arn1p, Arn2p, Enb1p, or Fui1p) are resistant to the drug, whereas *anr1*Δ, *arn2*Δ, *enb1*Δ single mutants or mutations in combination with the gene encoding iron permease Ftr1p do not confer a growth advantage.

We performed validation experiments to investigate the resistance of double mutants enriched in CGP to 1,10-phenanthroline (Fig. 7). Among the strains enriched in the CGP were *fui1*Δ, *arn1*Δ, *arn2*Δ, and *enb1*Δ strains. Fui1p is a high-affinity uridine permease, and the transporters Arn1p, Arn2p, and Enb1p have similar cargo specificity (ARN family transports siderophore-iron chelates, and Enb1p transports ferric enterobactin), whereas Ftr1p is a high-affinity iron permease, which is also involved in iron homeostasis. However, *ftr1*Δ strains were depleted in CGP assays. Even though Arn1p, Arn2p, Enb1p, and Ftr1p are iron transporters, only the transporters with specificity for large molecules, such as Arn1p, Arn2p, Enb1p, and Fui1p appear to contribute to 1,10-phenanthroline transport. Single transporter deletions or deletions in combination with *ftr1*Δ do not confer resistance probably due to redundancy. Only with the deletion of at least two of the four suggested transporters can we limit the compound’s uptake to confer measurable resistance. Our results demonstrate the power of the double mutant deletion library in identifying groups of transporters that contribute to the import of the test compound.

### Validation of transporter-mediated import routes of azoles.

**FIG 8:**
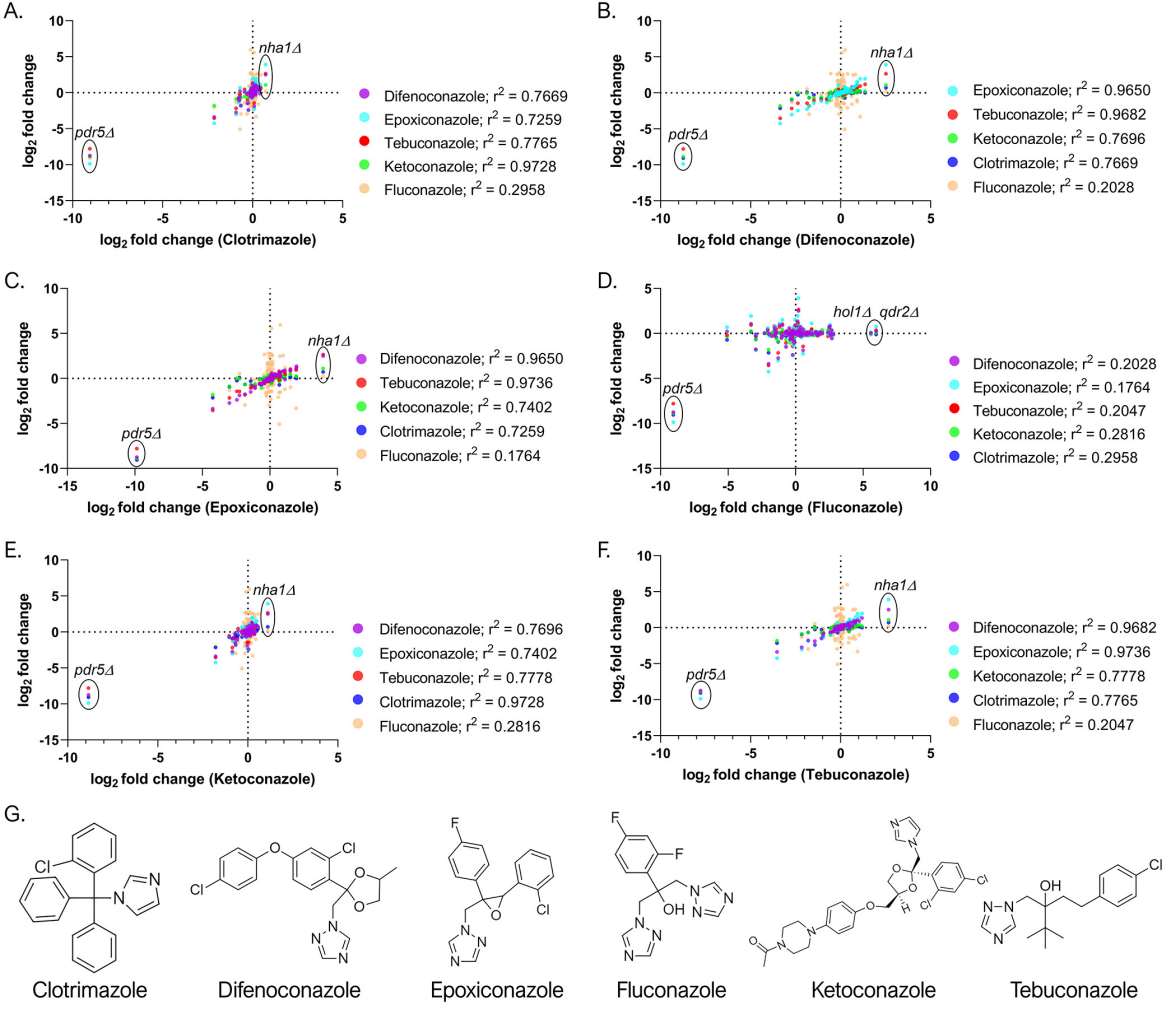
Relationship between azole influx and efflux transporters based on CGP results. The analysis shows a good correlation (*r*^2^ > 0.95) between proposed transport routes for agrochemical azoles (difenoconazole, epoxiconazole, and tebuconazole) members of the 1,2,4-triazole class and between the animal antifungals clotrimazole and ketoconazole (*r*^2^ = 0.9728), which are members of the imidazole class. (A to F) Correlation graphs with clotrimazole, difenoconazole, epoxiconazole, fluconazole, ketoconazole, and tebuconazole, respectively, in abscissa and other 5 in ordinate. (G) Chemical structure of the azole xenobiotics. Different colors identify the xenobiotic represented. Strains with the highest and lowest abundance were labeled for identification.

With the exception of fluconazole, azole compounds showed very consistent results with regard to their import and export routes (evident in the PCA plot [Fig. 6]). Azoles are antifungal agents that target the ergosterol (sterol) biosynthesis pathway by inhibiting lanosterol 14-alpha demethylase (a cytochrome P450), encoded by the *ERG11* gene in S. cerevisiae (20, 39, 40). When analyzing the correlation between the CGP results of six azole compounds, we found that three azole antifungals (difenoconazole, epoxiconazole, and tebuconazole) show very similar profiles of genes involved in the import and export of these compounds (*r*^2^> 0.95) (Fig. 8B, C, and F). These antifungal agrochemicals are members of the 1,2,4-triazole class and also present a halogenated benzene ring (two in difenoconazole and epoxiconazole; one in tebuconazole) (Fig. 8G). The azole antifungals of the imidazole class, clotrimazole and ketoconazole, are drugs for animal use, and they also have a good correlation between importers and exporters (*r*^2^ = 0.97) (Fig. 8A and E). Fluconazole did not show any correlation with the other azoles (Fig. 8D). This may be due to differences in its structure, as this compound bears an additional nitrogen-containing five-membered ring and a difluorophenyl group. These results indicate that there is a clear relationship between compound structures and the import/export routes revealed by our chemogenomic approaches. Previous studies suggested that the azoles use facilitated diffusion and that both parts of the molecule (the nitrogen-containing five-membered and the halogenated benzene rings) are essential for cell uptake (41–43). We have demonstrated that these chemical groups show strong correlation to the substrate profile of protein transporters involved in the influx and efflux of the xenobiotics.

CGP and low-throughput assays of azole compounds showed an interesting profile of putative import and export routes (Fig. 8 and Table 1), suggesting a number of carriers potentially responsible for azole uptake. The *nha1*Δ::kanMX deletion mutant, for example, was a top hit for five of the six azoles tested (CGP), and *nha1*Δ deletants were identified among the resistant colonies selected in a plate assay (low-throughput assay) for clotrimazole, ketoconazole, and difenoconazole (Table 1), supporting the possible involvement of this cation antiporter in the transport of these compounds. Itr1p, which is responsible for uptake of *myo*-inositol (44), is a putative import route, as *itr1*Δ strains are resistant to the triazoles difenoconazole, epoxiconazole, and tebuconazole in both assays (Table 1). In accordance with this finding, we observed that most of the double-deletion strains resistant to triazoles in the high-density plate assay bear the deletion of the *ITR1* gene (Fig. S4, Fig. S5, and Fig. S6). We also observed the resistance of the *itr1*Δ strain to the imidazoles clotrimazole and ketoconazole in the high-density assay (Fig. S7 and Fig. S8). This is in agreement with previous work (8) in which the deletion of *ITR1* conferred resistance to the compounds clotrimazole, ketoconazole, and fluconazole in plate experiments.

10.1128/mbio.03221-21.5 FIG S4 High-density assay with 308 double-deletion strains (in quadruplicate) in the presence of an inhibitory concentration of difenoconazole. The upper left panel shows a test plate containing difenoconazole (4 days of growth). The upper right panel shows a control plate with 2% DMSO (4 days of growth). The middle panel presents a plate map (::*nat*MX followed by ::*kan*MX), and the lower panel shows the z-score for each quadruplicate. Green squares correspond to scores 3*SD, and orange squares correspond to between 2*SD and 3*SD. The high-density assay presents the same DMSO 2% plate as the experiment control. Download FIG S4, TIF file, 2.4 MB. Copyright © 2021 Almeida et al. 2021 Almeida et al. https://creativecommons.org/licenses/by/4.0/ This content is distributed under the terms of the Creative Commons Attribution 4.0 International license.

10.1128/mbio.03221-21.6 FIG S5 High-density assay with 308 double-deletion strains (in quadruplicate) in the presence of an inhibitory concentration of epoxiconazole. The upper left panel shows a test plate containing epoxiconazole (4 days of growth). The upper right panel shows a control plate with 2% DMSO (4 days of growth). The middle panel presents a plate map (::*nat*MX followed by ::*kan*MX), and the lower panel shows the z-score for each quadruplicate. Green squares correspond to scores 3*SD, and orange squares correspond to between 2*SD and 3*SD. The high-density assay presents the same 2% DMSO plate as the experiment control. Download FIG S5, TIF file, 2.4 MB. Copyright © 2021 Almeida et al. 2021 Almeida et al. https://creativecommons.org/licenses/by/4.0/ This content is distributed under the terms of the Creative Commons Attribution 4.0 International license.

10.1128/mbio.03221-21.7 FIG S6 High-density assay with 308 double-deletion strains (in quadruplicate) in the presence of an inhibitory concentration of tebuconazole. The upper left panel shows a test plate containing tebuconazole (4 days of growth). The upper right panel shows a control plate with 2% DMSO (4 days of growth). The middle panel presents a plate map (::*nat*MX followed by ::*kan*MX), and the lower panel shows the z-score for each quadruplicate. Green squares correspond to scores 3*SD, and orange squares correspond to between 2*SD and 3*SD. The high-density assay presents the same 2% DMSO plate as the experiment control. Download FIG S6, TIF file, 2.4 MB. Copyright © 2021 Almeida et al. 2021 Almeida et al. https://creativecommons.org/licenses/by/4.0/ This content is distributed under the terms of the Creative Commons Attribution 4.0 International license.

10.1128/mbio.03221-21.8 FIG S7 High-density assay with 308 double-deletion strains (in quadruplicate) in the presence of an inhibitory concentration of clotrimazole. The upper left panel shows a test plate containing clotrimazole (4 days of growth). The upper right panel shows a control plate with 2% DMSO (4 days of growth). The middle panel presents a plate map (::*nat*MX followed by ::*kan*MX), and the lower panel shows the z-score for each quadruplicate. Green squares correspond to scores 3*SD, and orange squares correspond to between 2*SD and 3*SD. The high-density assay presents the same 2% DMSO plate as the experiment control. Download FIG S7, TIF file, 2.5 MB. Copyright © 2021 Almeida et al. 2021 Almeida et al. https://creativecommons.org/licenses/by/4.0/ This content is distributed under the terms of the Creative Commons Attribution 4.0 International license.

10.1128/mbio.03221-21.9 FIG S8 High-density assay with 308 double-deletion strains (in quadruplicate) in the presence of an inhibitory concentration of ketoconazole. The upper left panel shows a test plate containing ketoconazole (4 days of growth). The upper right panel shows a control plate with 2% DMSO (4 days of growth). The middle panel presents a plate map (::*nat*MX followed by::*kan*MX), and the lower panel shows the z-score for each quadruplicate. Green squares correspond to scores 3*SD, and orange squares correspond to between 2*SD and 3*SD. The high-density assay presents the same 2% DMSO plate as the experiment control. Download FIG S8, TIF file, 2.4 MB. Copyright © 2021 Almeida et al. 2021 Almeida et al. https://creativecommons.org/licenses/by/4.0/ This content is distributed under the terms of the Creative Commons Attribution 4.0 International license.

Evaluating the results obtained with the compound fluconazole, an azole of the triazole class, we observe a group of transporters, the deletion of which conferred a resistance phenotype in both plate and CGP approaches. The small- and large-scale screening showed that the following deletions may confer resistance to this compound and thus implicate the cognate transporters in the import of fluconazole: *qdr3*Δ, *tat1*Δ, *ady2*Δ, *adp1*Δ, *yor1*Δ, *arn1*Δ, *hxt4*Δ, *bor1*Δ, and *hol1*Δ. We did not perform further validation experiments for these transporters; however, the set of deletions that conferred resistance to fluconazole is different from those observed for other azoles and may contribute to the traffic of this compound.

### Itr1p is a putative azole importer.

**FIG 9:**
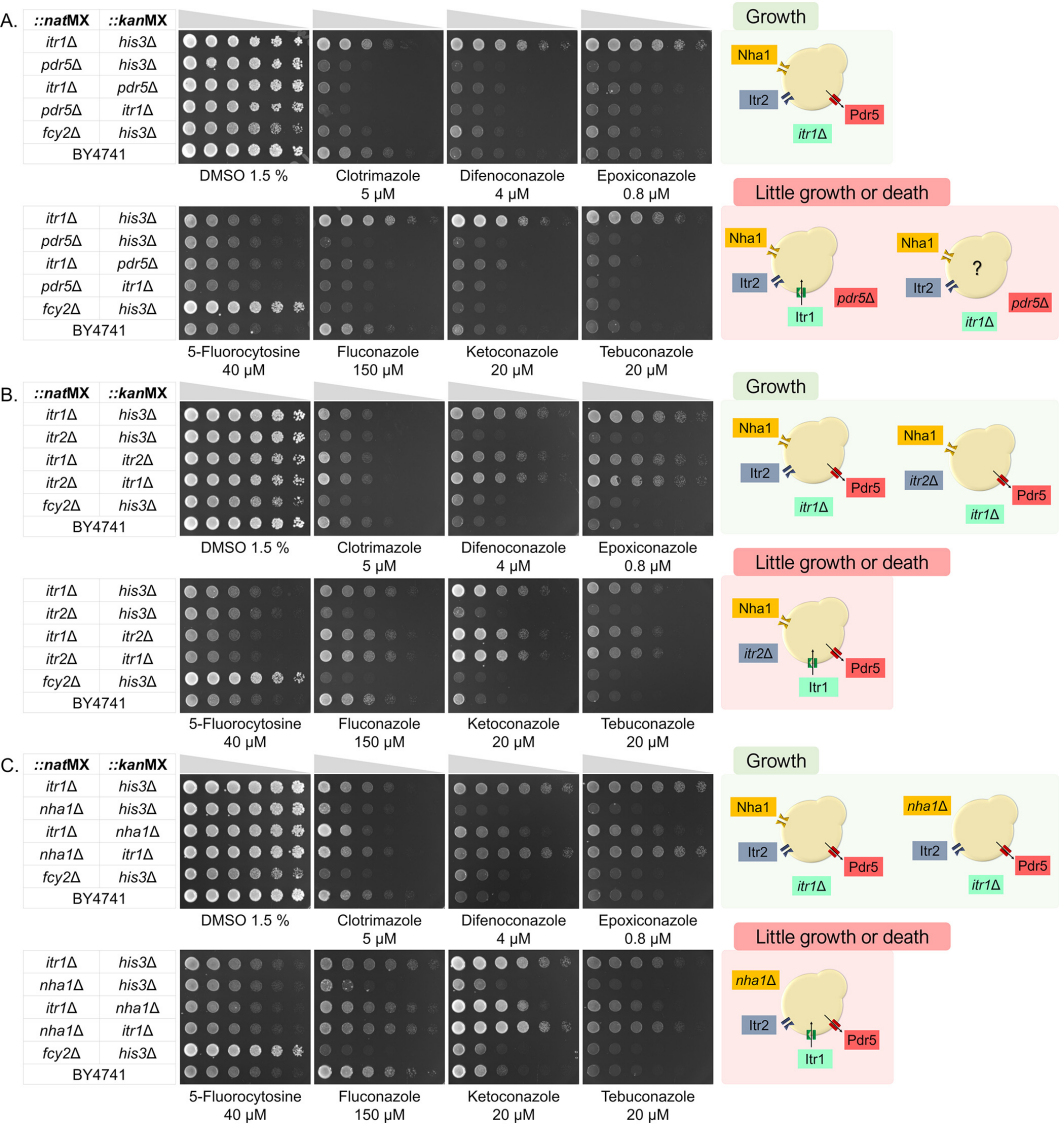
Evaluation of resistance or sensitivity phenotypes in double-deletion strains for putative importers and a known exporter of azole compounds. The panels present spot test of serial dilutions of double-deletion strains in the presence of the six azole compounds: clotrimazole, difenoconazole, epoxiconazole, fluconazole, ketoconazole, and tebuconazole. (A) The *itr1*Δ strain presented a resistance phenotype without the presence of a second transporter deletion and in the presence of Pdr5p. The *pdr5*Δ strain, as expected, presented a sensitivity profile, confirming its importance in azole efflux. However, with the *itr1*Δ *pdr5*Δ double mutant, a sensitive phenotype is observed, indicating a secondary uptake route. (B and C) The *itr2*Δ (*ITR1* paralog) strain and *nha1*Δ (experimental hit for azoles) strain, respectively, were tested as a secondary route. Plate assays indicate that deletion of these transporters does not confer resistance and may indicate that they are not involved in the uptake.

We investigated the role of Itr1p on azole import by evaluating the resistance phenotype conferred by the *itr1*Δ mutation, either alone or combined with *itr2*Δ, *nha1*Δ, *pdr5*Δ mutations. Small-scale assays confirmed the resistance phenotype (in plate assays and CGP) of *itr1*Δ strains to difenoconazole, epoxiconazole, ketoconazole, and tebuconazole in both solid and liquid media assays (Fig. 9 and data not shown). However, in the spot test, the *itr1*Δ mutant did not present a strong resistance phenotype in the presence of either clotrimazole or fluconazole.

Considering the important role of Pdr5p in azole efflux (19, 20), we investigated whether the resistance phenotype observed for the strain was due to the absence of the importer or to an activity of the exporter. We performed spot tests of BY4741 (wild type) and mutant strains in the presence of sublethal doses of the agrochemical triazoles, ketoconazole, clotrimazole, and fluconazole (Fig. 9A). Our results show that a cell containing all transporters (importers and exporters) is sensitive to these compounds and that export pumps cannot overcome their import. The *pdr5*Δ *his3*Δ double mutant was sensitive to azoles, corroborating previous findings that suggest a role for this multidrug transporter in the export of azole compounds (19, 20) (Fig. 9A). We found that the *itr1*Δ *pdr5*Δ double mutant strain is sensitive to azoles, which indicates that azoles accumulate inside the cell even in the absence of Itr1p, suggesting additional import routes for azole compounds (Fig. 9A). Hence, without an efficient efflux through Pdr5p, the cell is susceptible to the action of azoles even when its primary import route is absent.

Our approaches suggest a group of transporters that may contribute with Itr1p in azole uptake. In CGP and the high-density plate assay results, we observed that treatment with the agrochemical triazoles and ketoconazole selected a set of deletions that, in combination with *itr1*Δ, confer resistance phenotypes to the double mutants (Fig. S4, Fig. S5, Fig. S6, and Fig. S8), namely, *tpo5*Δ, *ftr1*Δ, *snq2*Δ, *smf1*Δ, *tpo1*Δ, *tna1*Δ, and *adp1*Δ. We also investigated *itr2*Δ, as *ITR2* is a paralog of *ITR1*, encoding a putative azole importer. However, in our plate assay (Fig. 9B), the *itr2*Δ mutant did not confer resistance to azoles. Nha1p was also suggested as a putative azole importer, considering that *nha1*::*kan*MX was the top CGP hit for azoles and also presented as a hit in the low-throughput assay (Table 1). However, validation studies with *nha1*Δ::*nat*MX showed no resistance profile (Fig. 9C), which may suggest that some *nha1*Δ::*kan*MX strains might carry additional mutations, for instance in genes specifying cytochrome P450s (45), that could be responsible for the observed phenotype.

## DISCUSSION

There is considerable controversy regarding the preferential mode of import of drugs into their target cells. The norm in the pharmaceutical industry is to design new drugs on the principle that they should be able to enter cells by passive diffusion through the plasma membrane lipid bilayer. However, this does not explain the fact that there are many efficacious drugs on the market whose physicochemical characteristics make it unlikely that they enter by this route (46). Furthermore, import by nonspecific passive diffusion does not explain the differences in drug import between different tissues and, in particular, the inability of many drugs to enter the brain (2). An alternative is that, for many drugs and other xenobiotics, the primary route of ingress is likely via protein carriers located in the plasma membrane that can affect either facilitated diffusion or active transport (2, 47). In order to evaluate the primary route of import of xenobiotics and identify any transporter(s) responsible for their entry into target cells, we assembled a collection of yeast strains that lacked the genes encoding either a single or a pair of membrane transporters that can be used to rapidly evaluate whether specific transporters were involved in the import or export of individual drugs by observing the resistance phenotypes resulting from gene loss.

The library used in this work contains double deletions of nonessential transporters and provides at least a twofold increase in resistant strains compared to the single-deletion library, thus enabling the identification of pairs of transporters involved in the entry of toxic compounds. Two strategies were employed for transporter identification: low-throughput (plate assay) and high-throughput assays (CGP), using a pool of the double mutant library, combined with validation assays with isolated strains. The low-throughput approach clearly yields valuable and verifiable results; however, it is laborious and demands high sampling to obtain significant data. Alternatively, we employed high-throughput assays, where pools of double mutant transporter deletion strains were grown in competition and the relative contribution of each transporter to the import or export of a given test compound was inferred from the resistance (enrichment) or sensitivity (depletion) phenotypes conferred by their deletion. CGP provided quantitative clues to the relative contribution of each transmembrane protein to the transport of different compounds across the plasma membrane.

Out of 21 compounds tested in CGP in this work, 14 selected for significantly enriched strains, among which we were able to observe the same deletion strains as hits from the low- and high-throughput screens. For example, CGP, low-throughput, and high-density assays suggested putative yeast plasma membrane transporters for cytotoxic compounds and indicated that the *myo*-inositol transporter Itr1p plays a significant role in the uptake of azoles (both triazoles and imidazoles), which is in agreement with previous work that indicated the entry of azoles into the cell is via facilitated diffusion in an ATP-independent process (41–43). CGP also provided consistent resistance and sensitivity data for the triazole agrochemicals and the clinically important imidazoles, identifying not just influx carriers but also the efflux pumps that may export these xenobiotics. We were able to identify the ABC multidrug resistance transporter Pdr5p (18) as the exporter of the six azole compounds tested, a result consistent with previous studies (19, 20). Given the importance of efflux carriers in drug resistance (48), characterizing the specificity of these carriers could contribute to the development of drugs refractory to transport via ABC transporters or the development of therapies in which the primary drug is used in combination with an export pump inhibitor.

Competition assays between azoles and *myo-*inositol did not reveal azole resistance (data not shown), and the *pdr5*Δ *itr1*Δ double mutant was also sensitive to azole treatment, indicating that an alternative import route is used in the absence of Itr1p. Furthermore, in spite of the evidence suggesting the import of azoles by Itr1p, the deletion of *ITR2*, a paralog of *ITR1* generated by the whole-genome duplication event, did not provide a resistance profile for the strain. Both transporters are responsible for uptake of *myo*-inositol and have high sequence homology (44); however, our screen indicates that Itr2p is unlikely to be involved in azole import. Itr1p is described as the major transporter of *myo*-inositol, and Itr2p plays only a minor role (44), which may explain the differences in the resistance profiles observed between *itr1*Δ and *itr2*Δ deletion strains.

In a search for transporters that may be either secondary azole transporters or have an indirect effect on these drugs’ efficacy, we focused on *nha1*Δ, which was a recurrent hit. Nha1p is a Na^+^/K^+^ antiporter that acts in the active export of alkaline cations (Li^+^, Na^+^, K^+^, and Rb^+^) (31–33). It was not immediately obvious how Nha1p could be directly responsible for azole import. Hence, we performed validation experiments using the *nha1*Δ::*nat*MX *his3*Δ::*kan*MX, *itr1*Δ::*nat*MX *nha1*Δ::*kan*MX, and *nha1*Δ::*nat*MX *itr1*Δ::*kan*MX double mutants (Fig. 9C). While the *nha1*Δ::*kan*MX deletant was resistant to azoles in both low- and high-throughput assays (we were able to track only the *kan*MX barcodes), we did not observe any resistance profile for *nha1*Δ::*nat*MX *his3*Δ::*kan*MX, a strain bearing the Itr1 transporter. Hence, the presence of *nha1*Δ as a top hit may be due to additional mutations in the strain carrying *nha1*Δ::*kan*MX.

Our library consists of approximately 14,000 strains constructed by crossing 122 transporter gene deletions (*kan*MX marker with barcodes) with 120 transporter gene deletions (*nat*MX marker without barcodes). Considering all the combinations, we have double deletions of importers and exporters that can improve the identification of transport routes. We believe that this strategy for identifying which transporters are involved in the transport of specific compounds could be improved by performing pairwise crosses of all nonessential transporter gene deletions (including genes not represented in our library) barcoded in both alleles to better represent all nonessential import routes and facilitate the identification of transporter pairs working in xenobiotic import. With the development of new strategies for mapping compound import and export routes, we aim to contribute to our understanding of resistance mechanisms, which is critical for the design of drugs with continued efficacy. Furthermore, the knowledge of transporter substrate specificity may allow the design of prodrugs with enhanced targeting to the cell type of interest. Hence, we are convinced that our double-deletion library is an invaluable tool for the design of more specific and efficient therapies.

## MATERIALS AND METHODS

### Media.

The following media were used for the construction of the double mutant collection by SGA (49–51): YPD (2% Bacto peptone, 1% yeast extract, 2% glucose, 2% agar) with G418 (200 mg/liter) or clonNAT (100 mg/liter), enriched sporulation medium (20 g/liter agar, 10 g/liter potassium acetate, 1 g/liter yeast extract, 0.5 g/liter glucose, 12.5 mg/liter histidine, 12.5 mg/liter lysine, 12.5 mg/liter uracil, 62.5 mg/liter leucine), selective YNB medium (6.7 g/liter yeast nitrogen base with ammonium sulfate and without amino acids, 50 mg/liter canavanine, 50 mg/liter thialysine, 150 mg/liter leucine, 40 mg/liter uracil, 40 mg/liter methionine, 2% glucose), and YNB/MSG medium (1.7 g/liter yeast nitrogen base without ammonium sulfate and without amino acids, 1 g/liter monosodium glutamic acid, 100 mg/liter clonNAT, 200 mg/liter G418, 50 mg/liter canavanine, 50 mg/liter thialysine, 150 mg/liter leucine, 40 mg/liter uracil, 40 mg/liter methionine, 2% glucose).

Drug sensitivity assays for determination of the inhibitory concentrations of xenobiotics and for selection of resistant strains were performed in YNB+Sc medium (6.7 g/liter yeast nitrogen base with ammonium sulfate and without amino acids, complete amino acid supplement, 2% glucose), with or without 2% Bacto agar.

### Commercial xenobiotics.

We selected commercial xenobiotics, including agrochemicals and drugs for both human and animal use, and prepared 10 mM stock solutions (20 mM for artesunate; 40 mM for dl-4-hydroxy-3-methoxymandelic acid and tamoxifen) in 100% DMSO of the compounds purchased from Sigma-Aldrich (Merck Group) (see Table S1 in the supplemental material). We selected 5-fluorocytosine (catalog number F7129; Sigma-Aldrich, Merck Group) as a positive control for the assays, as the deletion of *FCY2* (YER056C; encoding the purine-cytosine permease) is well characterized and provides a resistant phenotype to this compound (8, 52).

### Strains.

A double mutant S. cerevisiae library was constructed by crossing single mutant strains in the BY741 (*MAT****a*** *his3*Δ*1 leu2*Δ*0 met15*Δ*0 ura3*Δ*0*) background (53) with single mutant strains in the Y7092 (*MATα can1*Δ::*STE2pr-Sp_his5 lyp1*Δ *his3*Δ*1 leu2*Δ*0 ura3*Δ*0 met15*Δ*0*) background (50, 51). In BY4741 genetic background, one of the following plasma membrane transporter-encoding genes was replaced with the antibiotic resistance marker *kan*MX, flanked by unique sequences (genetic barcodes) identifying deletions in each of the following open reading frames: YAL067C, YBL042C, YBR008C, YBR021W, YBR043C, YBR068C, YBR069C, YBR180W, YBR294W, YBR295W, YBR296C, YBR298C, YCL025C, YCR010C, YCR011C, YCR028C, YCR098C, YDL199C, YDR011W, YDR046C, YDR345C, YDR384C, YDR387C, YDR406W, YDR497C, YDR508C, YDR536W, YEL063C, YEL065W, YER056C, YER145C, YER166W, YFL011W, YFL040W, YFL050C, YFL055W, YGL077C, YGL114W, YGL255W, YGR055W, YGR121C, YGR138C, YGR217W, YGR224W, YGR260W, YGR281W, YGR289C, YHL016C, YHL040C, YHL047C, YHR092C, YHR094C, YHR096C, YIL013C, YIL088C, YIL120W, YIL121W, YJL093C, YJL129C, YJL212C, YJL214W, YJR040W, YJR054W, YJR152W, YKL174C, YKL217W, YKR039W, YKR050W, YKR103W, YKR106W, YLL028W, YLL043W, YLL048C, YLL052C, YLL061W, YLR081W, YLR092W, YLR130C, YLR138W, YLR237W, YML047C, YML116W, YML123C, YMR011W, YMR177W, YMR243C, YMR279C, YMR319C, YNL065W, YNL142W, YNL268W, YNL270C, YNL275W, YNL291C, YNL318C, YNR002C, YNR055C, YNR056C, YNR072W, YOL020W, YOL103W, YOL122C, YOL158C, YOR011W, YOR071C, YOR153W, YOR192C, YOR202W, YOR273C, YOR306C, YOR328W, YOR348C, YPL036W, YPL058C, YPL092W, YPL265W, YPL274W, YPR124W, YPR138C, YPR156C, YPR192W, YPR198W, and YPR201W (54). In the Y7092 background, the same plasma membrane transporter-encoding genes (except YEL063C, YHR096C, and YOR202W) were replaced with the *nat*MX marker (49). In the library construction, strains containing deletions of genes not related to transport (YAL060W, YDR073W, and YIR002C) were added as negative controls.

### Construction of the transporter double mutant library by synthetic genetic array.

The construction of the transporter double mutant collection library was performed essentially as described by Tong and coworkers (49–51). Briefly, 122 transporter-encoding gene deletion strains (plus the control strain with YOR202W deletion), in the BY741 background (53) were grown in 384-colony arrays (pinned using the Singer Rotor HAD, Singer Instruments, UK) on YPD with G418 (200 mg/liter) for 1 day at 30°C. In parallel, 120 strains in the Y7092 background (50, 51) were grown in 384-colony arrays on YPD with clonNAT (100 mg/liter) for 1 day at 30°C. Strains of the opposite mating type were then pinned onto fresh YPD plates and allowed to mate at room temperature for 24 h. They were then pinned onto YPD+G418+clonNAT and incubated at 30°C for 2 days to select for diploid cells. The diploids were then pinned onto enriched sporulation medium and incubated at room temperature for 5 to 10 days. The *MAT****a*** meiotic progeny were selected by pinning the sporulated strains onto YNB and incubated at 30°C for 2 days. Transporter double mutant *MAT****a*** strains were then selected by pinning onto YNB and incubating at 30°C for 2 days. This last step was repeated to ensure that all strains were indeed double mutants. Double mutants were replicated into 384-well plates with YPD plus 15% (vol/vol) glycerol and stored at −80°C. Double mutants were also pooled in the ratio of 1:1:1:1:…:1 and stored in YPD plus 15% (vol/vol) glycerol in 5-ml aliquots for competition experiments (library pools). The transporter deletion library constructed during the current study will be deposited in EUROSCARF.

### Determination of inhibitory concentrations of commercial xenobiotics.

S. cerevisiae BY4741 was inoculated into 5 ml of fresh YNB+Sc and grown overnight at 30°C with agitation. Then the culture was diluted to an optical density at 595 nm (OD_595_) of 0.1 in 70 μl of YNB+Sc containing different dilutions of each xenobiotic. The xenobiotics were tested at the following concentrations: 200 μM, 100 μM, 40 μM, 20 μM, 8 μM, 4 μM, 1.6 μM, 0.8 μM, 0.32 μM, and 0.16 μM. Controls containing 2% and 1% DMSO (vol/vol) were also tested. Cultures were prepared in quadruplicate in 384-well flat-bottom plates and incubated at 30°C, with linear shaking (700 rpm) in the CLARIOstar (BMG Labtech) plate reader, for 30 h with OD_595_ measurements every 10 min. Curves derived from the growth data were smoothed based on the moving average of the 15 closest measurements, and the growth score was calculated by multiplying the yield (maximum OD – minimum OD) by the maximum slope of the curve and dividing by the time taken to reach the maximum slope, using the data analysis software MARS (BMG Labtech). Nonlinear regression for IC_90_ definition was performed using GraphPad Prism version 8.0.0 for Windows, GraphPad Software, San Diego, California, USA.

Spot assays were performed in petri dishes (90 × 15 mm) containing YNB+Sc with inhibitory concentrations of a xenobiotic, as defined by growth assays in liquid cultures, with equivalent volumes of DMSO in negative-control plates. Serial dilutions (1:5 dilution) of BY4741, single mutant and double mutant library pool and selected single mutant strains were spotted onto control or xenobiotic-containing plates for selective inhibition verification and, for definition of inhibitory concentrations, onto solid media using 48-pin replicators (Sigma-Aldrich, Merck Group). Plates were incubated at 30°C for 2 to 4 days, and images were registered with ChemiDoc MP (Bio-Rad).

### Selection of resistant strains.

Assays were performed on petri plates (90 × 15 mm) containing YNB+Sc agar with inhibitory concentration of xenobiotics. Onto these plates, approximately 10^3^, 10^4^, 10^5^, and 10^6^ CFU of BY4741 or the double mutant library pool were plated. Plates containing YNB+Sc with DMSO were used as plating controls. After 2 days of incubation at 30°C, growth was registered with ChemiDoc MP (Bio-Rad), and resistant colonies from library pool plates were picked and transferred to fresh nonselective plates.

### Identification of resistant strains.

Genomic DNA was prepared as described by Lõoke et al. (55). Cells from resistant colonies were lysed by resuspension into 100 μl of 200 mM lithium acetate (LiAc) with 1% sodium dodecyl sulfate (SDS), followed by incubation at 70°C for 15 min. DNA was precipitated by the addition of 300 μl of 100% ethanol, followed by briefly vortexing and centrifugation for 3 min at 15,000 × *g*. Pellets were washed with 200 μl of 70% (vol/vol) ethanol, centrifuged for 3 min at 15,000 × *g*, and air dried. Genomic DNA (gDNA) pellets were dissolved in 100 μl of ultrapure water, followed by centrifugation for 15 s at 15,000 × *g*. PCRs using *Taq* DNA polymerase P1011 (Sinapse Inc.) were prepared following the manufacturer’s instructions. Barcode amplification was performed using primers pairs U1 forward 5′-GATGTCCACGAGGTCTCT-3′ with *kan*MX reverse 5′-CATCATTGGCAACGCTAC-3′ (upstream barcode) or *kan*MX forward 5′-CTCCTTCATTACAGAAACGG-3′ with D1 reverse 5′-CGGTGTCGGTCTCGTAG-3′ (downstream barcode). PCR products were purified using the E.Z.N.A. gel extraction kit (Omega Bio-tek) and sequenced by Sanger Sequencing. For upstream barcode sequencing, primer pTEF seq reverse 5′- CGACAGTCACATCATGCC-3′ was used, and for downstream barcode sequencing, primer *kan*MX forward was used. Sequencing was performed at Myleus Biotechnology using capillary electrophoresis (ABI3730) using POP7 polymer and BigDye v3.1. Sequence analyses and barcode identification were performed based on the *Saccharomyces* Genome Deletion Project barcode list.

### Chemical genomic profiling.

Determination of the inhibitory concentration of xenobiotics for transporter deletion strains was accomplished by growth curve assays of library pools. These assays were performed in liquid media in 48-well flat-bottom plates with incubation at 30°C, with 500 rpm double orbital shaking in the CLARIOstar (BMG Labtech) plate reader, for 48 h with OD_595_ measurements every 10 min. Concentrations selected for chemical genomic profiling were those that inhibited growth of transporter deletion pools by approximately 80% while allowing the culture to reach the stationary phase in 24 h.

The transporter double-deletion library pool was grown in YNB+Sc for 12 h at 30°C with agitation. Pools were then diluted to an OD_600_ of 0.1 in 500 μl of YNB+Sc containing the xenobiotic compounds in 48-well flat-bottom plates (samples were prepared in quadruplicate). After 24 h of growth at 30°C with agitation (ca. 5 generations), OD_595_ was measured, and cultures were diluted 20× in 500 μl of fresh media containing the xenobiotic and allowed to grow for 12 h (ca. 10 generations) under the same conditions. The dilution procedure was repeated, and cultures were allowed to grow until stationary phase (ca. 15 generations). Cell pellets were collected, and genomic DNA was extracted using the Wizard Genomic DNA purification kit. Upstream barcodes were PCR amplified with U1 and U2 primers containing Illumina preadapters for multiplex barcode sequencing with Illumina HiSeq2500 platform by the University of São Paulo Functional Genomics Center.

The quality of the generated reads was analyzed with the FastQC (version 0.11.7) (56) and MultiQC (version 1.6) (57) software before and after removal of primers and adapters performed with the Cutadapt tool (version 1.26) (58). DADA2 (version 1.9.1) (59) was employed to infer amplicon sequencing variants (ASVs) by trimming and discarding low-quality reads, correcting sequencing errors (denoising) and merging read pairs. Since it is known that several barcodes have sequences different from those that were originally described (60), ASVs that did not match any previously described barcode were assigned to the most similar barcode sequencing if the Levenshtein distance was equal to or less than 2. If two or more ASVs matched the same barcode, the read counts of those ASVs were combined. With the DESeq2 package (version 1.20.0) (61), the normalization of counts and the assessment of the differential abundance of barcodes between samples treated against untreated controls were performed. For principal-component analysis (PCA), the barcode count matrix was transformed using the rlog function to standardize the abundance variance between the different barcodes. Differentially abundant strains were identified using a maximum likelihood ratio test, and normalization between samples was done by the library size factor method (62). Differentially abundant barcodes in treated versus control comparisons were considered significant for the *P* value adjusted for multiple tests (padj) by the Benjamini-Hochberg method (≤0.1) and *P* value less than 0.001. For the analysis, we used thresholds of log_2_ fold change at ≥0.5 (for resistant strains) and ≤−0.5 (for sensitive strains). Correlation from 116 genes presented in all azole data from CGP was performed using GraphPad Prism version 8.0.0 for Windows, GraphPad Software, San Diego, California, USA. CGP data sets with differential abundance of barcodes are presented in Data Set S1 in the supplemental material.

### High-density plate assays for transporter validation.

Based on chemical genomic results, 308 double-transporter-deletion mutants were selected for validation (padj ≤ 0.1; *P* value ≤ 0.001; log_2_ fold change ≥ 0.5). These mutants were inoculated in 50 μl of YNB+Sc liquid medium in a 384-well plate and grown at 30°C until saturation (∼36 h). Using the Rotor HDA (Singer Instruments, UK), cultures were stamped in quadruplicate (1,536 spots) on plates containing solid YNB+Sc medium at inhibitory concentrations (1× and 2×) of the test compounds. Cultures were incubated for 2 days at 30°C and on subsequent days at room temperature (∼25°C). Plate photos were registered with ChemiDoc MP (Bio-Rad).

Quantification of the growth of strains was performed by analyzing the images in .jpg format using a python script. The OpenCV package contour detection module for python was used to delimit the plate and some colonies in the image. Based on this, the identification of the 1,536 spots and delimitation of the columns and lines of the plate was performed. Voids (16 spots without culture inoculation; four corners of the plate) and wild-type 288 spots (BY4741; plate edge) were identified. For each spot, a quadrangular cut-out of a fixed area was delimited. To estimate the growth in the spot, the pixel values in this cut-out were averaged, considering the black and white scale (values from 0 to 255). For each strain, the median of the four values was calculated according to the plate map. The z-score was calculated using median values, according to the median of a sample minus the mean of all median values, divided by the standard deviation of all median values. Strains that varied by more than 3 standard deviations (3*SD) from the mean were considered resistant.

### Small-scale validation assays.

Gene deletion of strains selected for validation were confirmed by PCR with “A_confirmation_primer” (*Saccharomyces* Genome Deletion Project) and *kan*MX reverse 5′-CATCATTGGCAACGCTAC-3′ (for *kan*MX gene deletion) or *nat*MX reverse 5′-AAGACGGTGTCGGTGGTG-3′ (for *nat*MX gene deletion). Overnight cultures in YNB+Sc were serially diluted in a 96-well plate and stamped using a replica plater with 48 pins (Sigma-Aldrich, Merck Group) in petri dishes with YNB+Sc solid medium containing inhibitory concentrations of xenobiotics. Cultures were incubated for 2 days at 30°C and on subsequent days incubated at room temperature (∼25°C). Plate photos were registered with ChemiDoc MP (Bio-Rad). Growth curves and nonlinear regression of selected deletants were performed as described for IC_90_ determination.

### Data availability.

High-throughput sequencing data of the CGP screens have been deposited in the NCBI Short Read Archive (SRA) under BioProject accession no. PRJNA718573 (BioSample accession nos. SAMN18541664 to SAMN18541685).
