# Supplementary material for: Yeast Double Transporter Gene Deletion Library for Identification of Xenobiotic Carriers in Low or High Throughput
Source: mBio. 2021 Dec 14;12(6):e03221-21. doi: 10.1128/mbio.03221-21 (PMC8669479; doi:10.1128/mbio.03221-21)
Supplement: TABLE S1 [file mbio.03221-21-st001.pdf]

Supplemental Table S1. **Xenobiotics used in this study.** *Compound name, catalogue numbers and chemical properties.*

Compound	Catalogue number (Sigma-Aldrich)	Hydrogen Bond Donor Count*	Hydrogen Bond Acceptor Count*	Molecular Weight (g/mol)*	XLogP3 (-AA)*
1,10-Phenanthroline	131377	0	2	180.20	1.8
2-Aminobenzimidazole Pestanal ®	31189	2	2	133.15	0.9
2-Hydroxybiphenyl Pestanal ®	45529	1	1	170.21	3.1
3,4-Dichloroisocoumarin	D7910	0	2	215.03	3.1
8-Hydroxyquinoline	H6878	1	2	145.16	2
Aldrin Pestanal ®	36666	0	0	364.90	4.5
Ammonium pyrrolidinedithiocarbamate	P8765	1	2	164.30	-
Artesunate	A3731	1	8	384.40	2.5
Cantharidin	C7632	0	4	196.20	0.6
Captan Pestanal ®	32054	0	3	300.60	2.4
Carbendazim	378674	2	3	191.19	1.5
Chlorothalonil Pestanal ®	36791	0	2	265.90	2.9
Cisplatin	PHR1624	2	2	300.00	-
Clotrimazole	C6019	0	1	344.80	5
Dazomet Pestanal ®	45419	0	3	162.30	1.3
Dichloran Pestanal ®	45435	1	3	207.01	2.9
Difenoconazole Pestanal ®	36531	0	5	406.30	4
DL-4-Hydroxy-3-Methoxymandelic Acid	H0131	3	5	198.17	-0.2
Epoxiconazole Pestanal ®	36848	0	4	329.80	3.2
Fluconazole	PHR1160	1	7	306.27	0.4
Fludioxonil Pestanal ®	46102	1	5	248.18	2.6
Fuberidazole	PS2059	1	2	184.19	2.2
Iprobenfos Pestanal ®	45814	0	4	288.34	3.2

Iprodione Pestanal ®	36132	1	3	330.16	3.1
Irgasan	72779	1	2	289.50	5
Ketoconazole	PHR1385	0	6	531.40	4.3
Mancozeb Pestanal ®	45553	4	8	541.10	-
N-Phenylanthranilic Acid	144509	2	3	213.23	4.4
Pencycuron Pestanal ®	31118	1	1	328.80	4.8
Tamoxifen	85256	0	2	371.50	7.1
Tebuconazole Pestanal ®	32013	1	3	307.82	3.8
Tunicamycin	T7765	11	16	816.90	-0.3

*data obtained from Pubchem (Kim S, Chen J, Cheng T, Gindulyte A, He J, He S, Li Q, Shoemaker BA, Thiessen PA, Yu B, Zaslavsky L, Zhang J, Bolton EE, Nucleic Acids Res 49:D1388–D1395, 2021, doi: 10.1093/nar/gkaa971).
