# Supplementary figures and images for: Yeast Double Transporter Gene Deletion Library for Identification of Xenobiotic Carriers in Low or High Throughput
Source: mBio. 2021 Dec 14;12(6):e03221-21. doi: 10.1128/mbio.03221-21 (PMC8669479; doi:10.1128/mbio.03221-21)

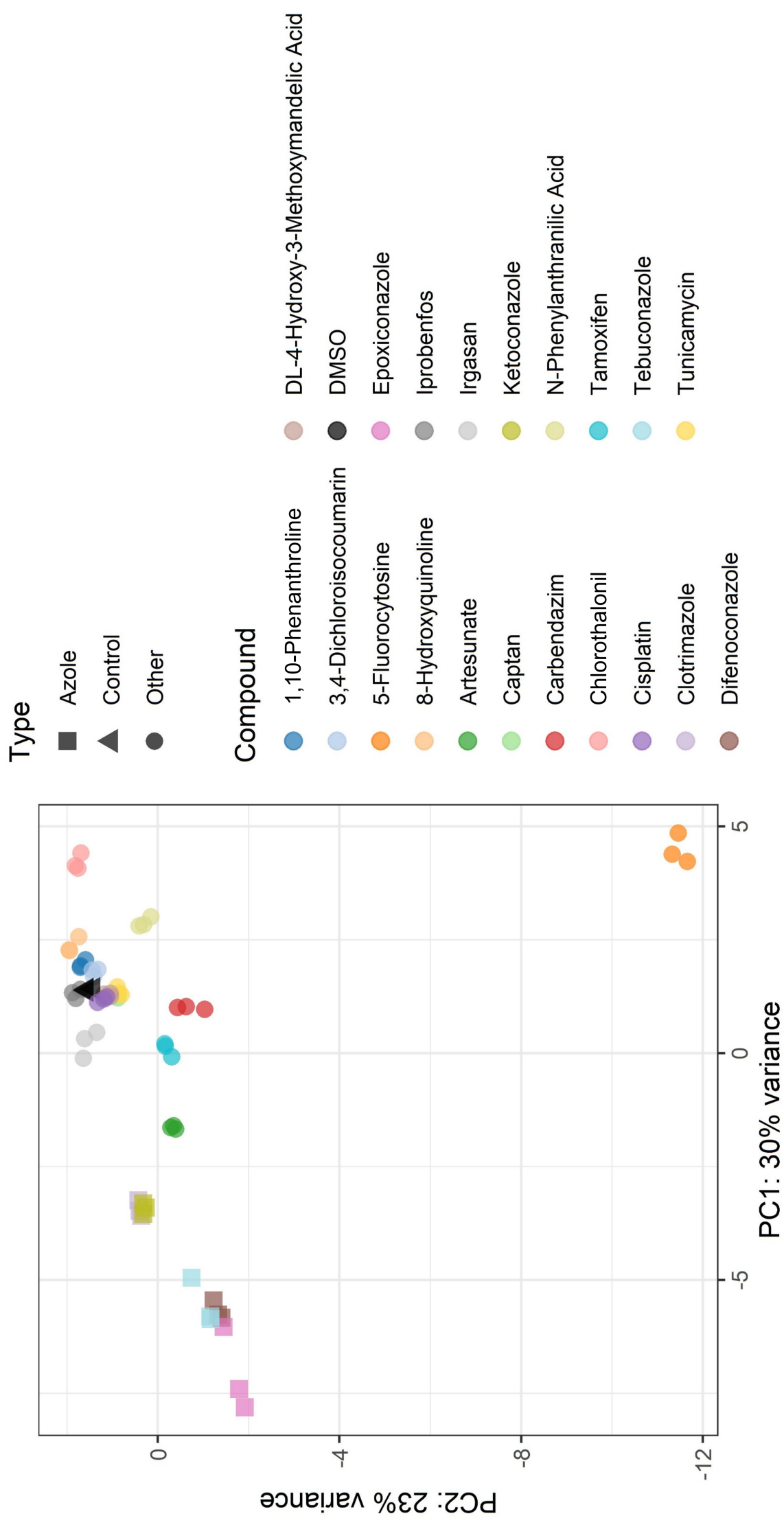

Supplement: FIG S1 [file mbio.03221-21-sf001.pdf]

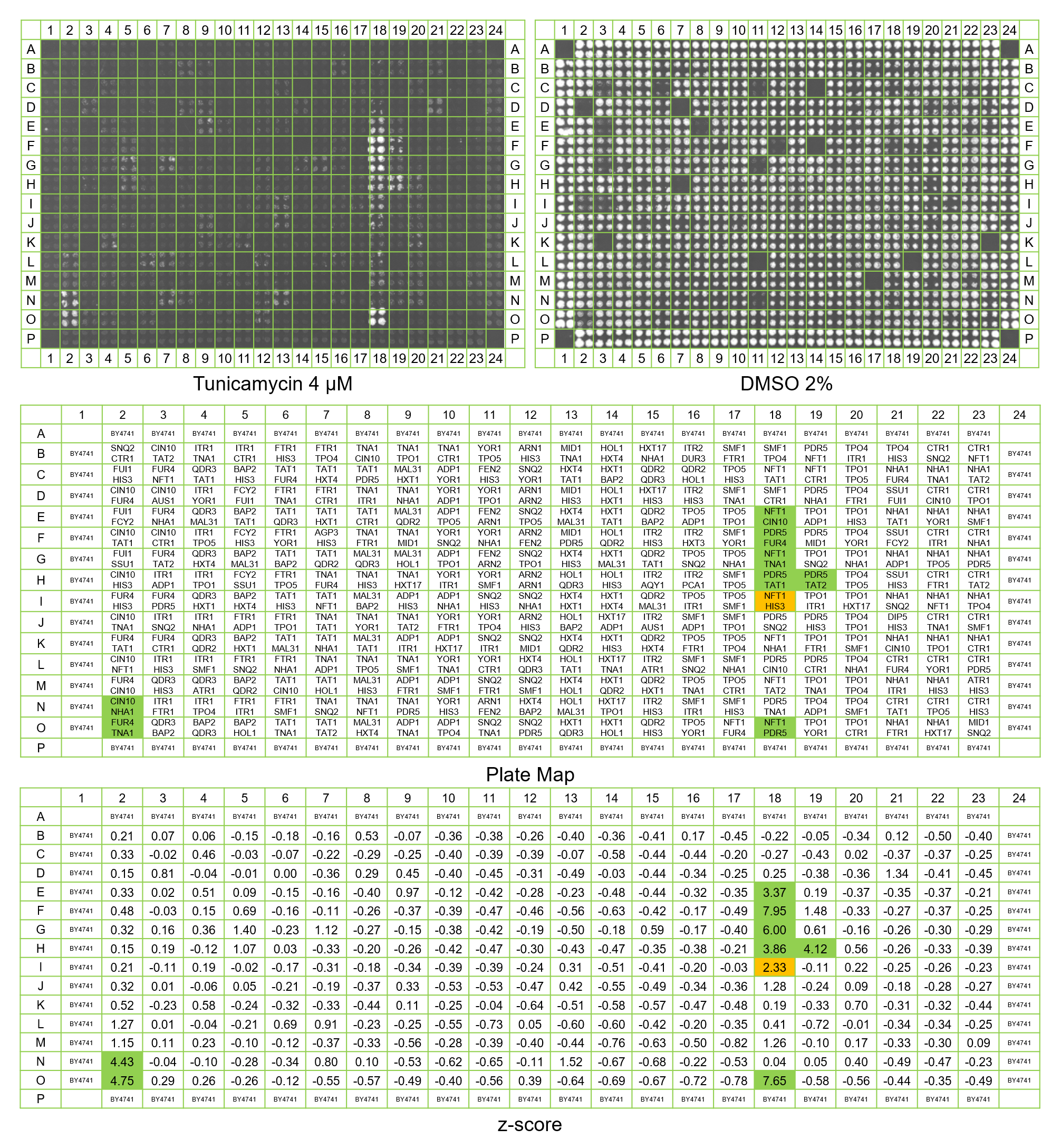

Supplement: FIG S2 [file mbio.03221-21-sf002.tif]

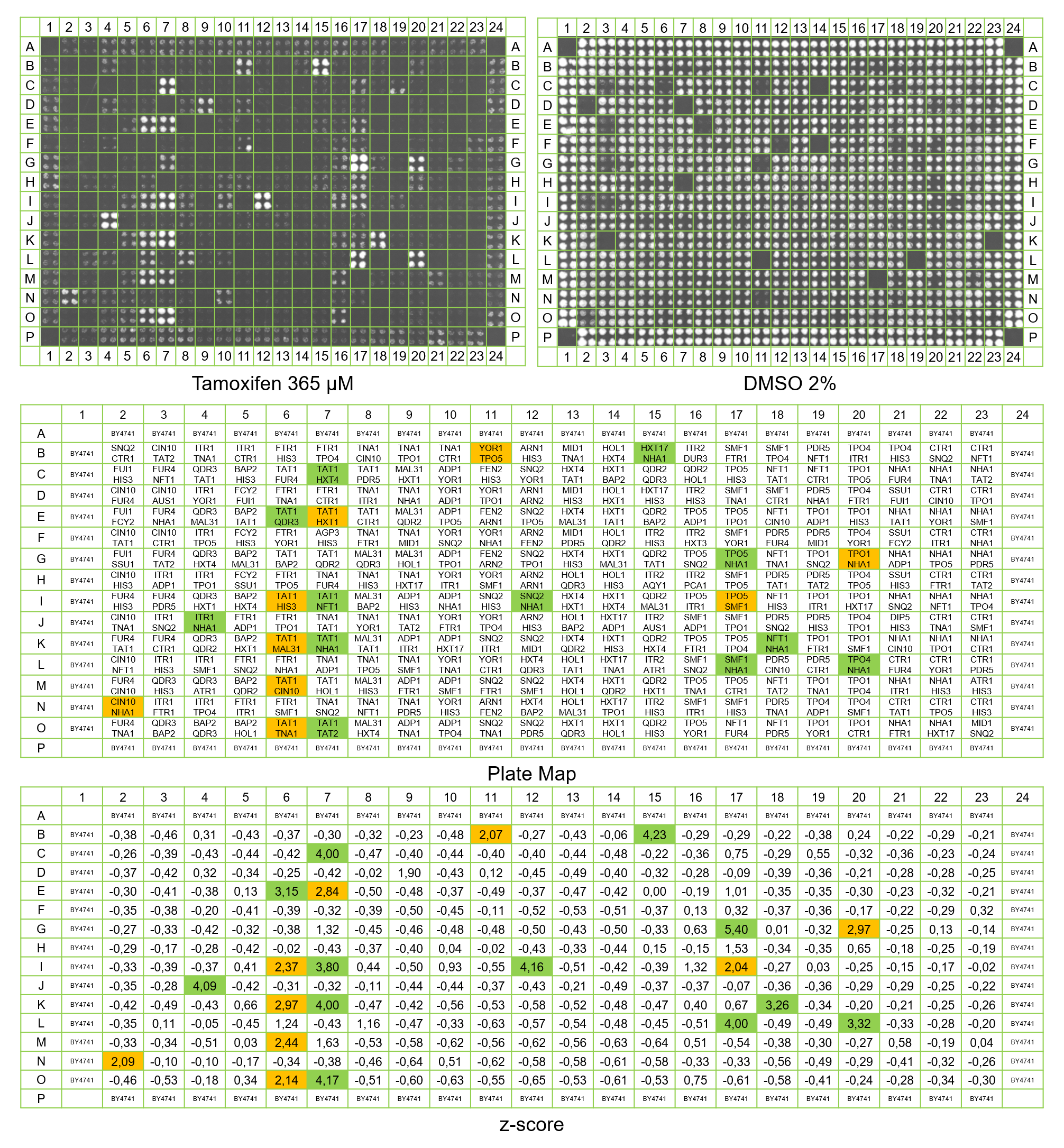

Supplement: FIG S3 [file mbio.03221-21-sf003.tif]

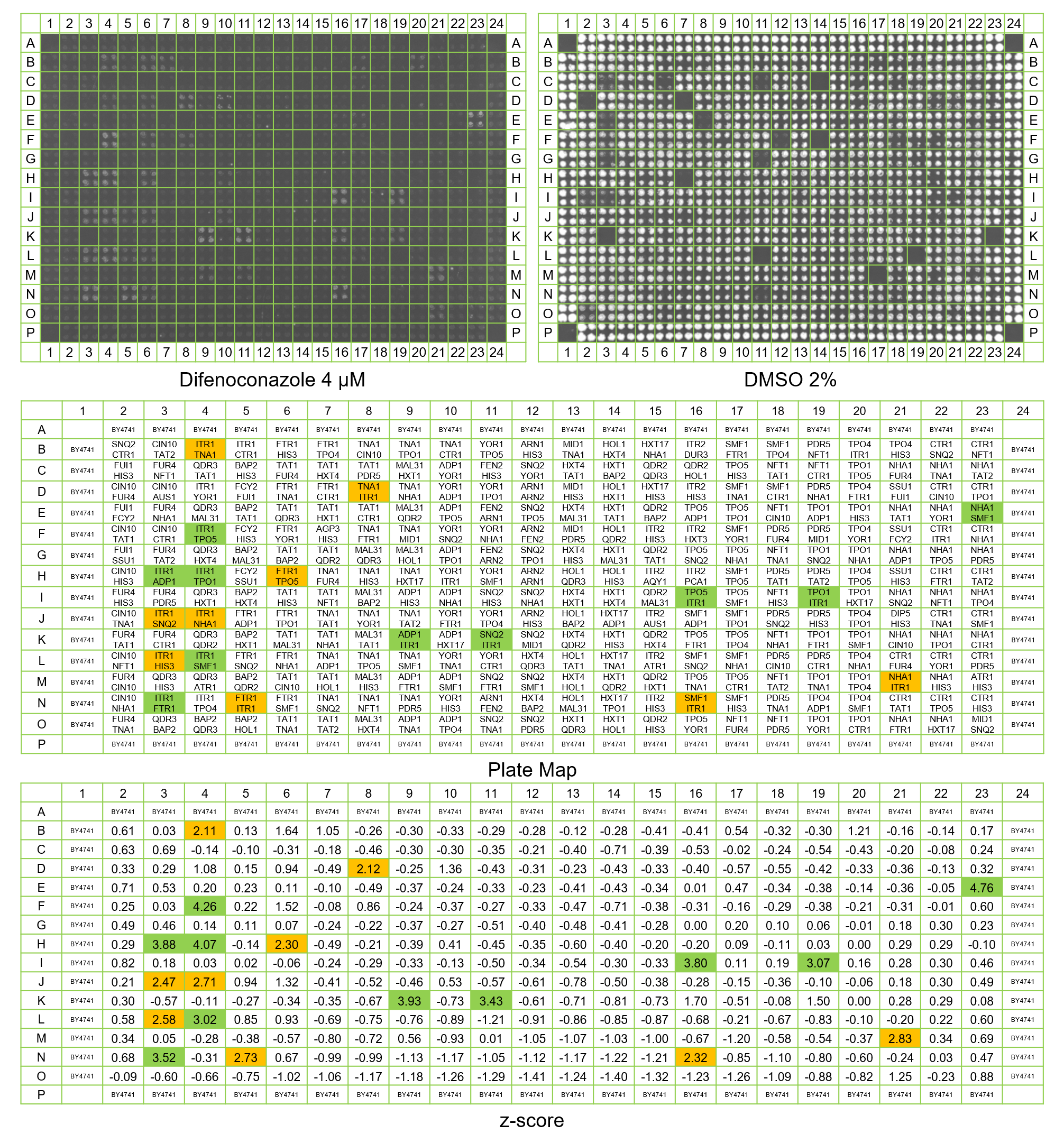

Supplement: FIG S4 [file mbio.03221-21-sf004.tif]

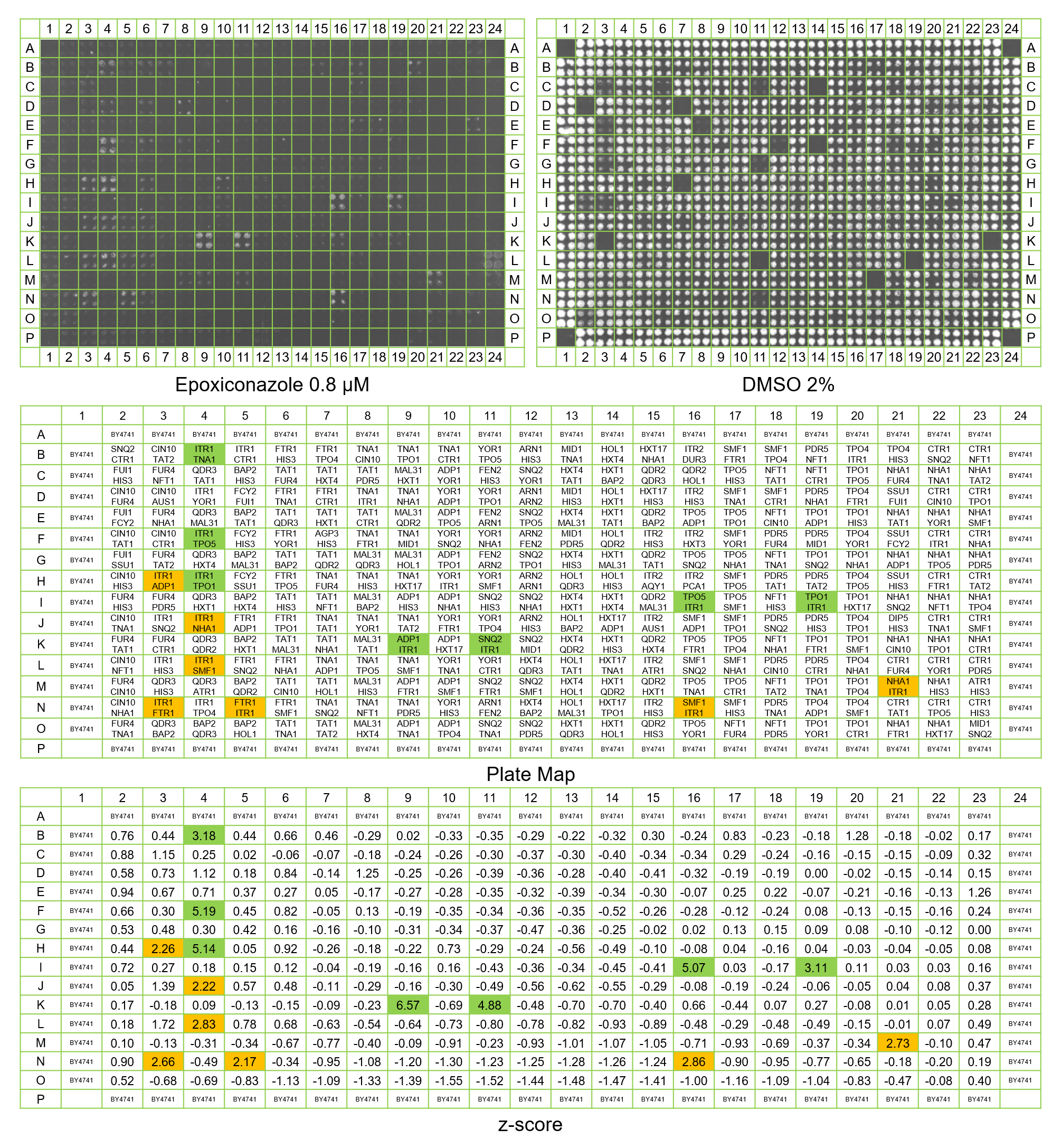

Supplement: FIG S5 [file mbio.03221-21-sf005.tif]

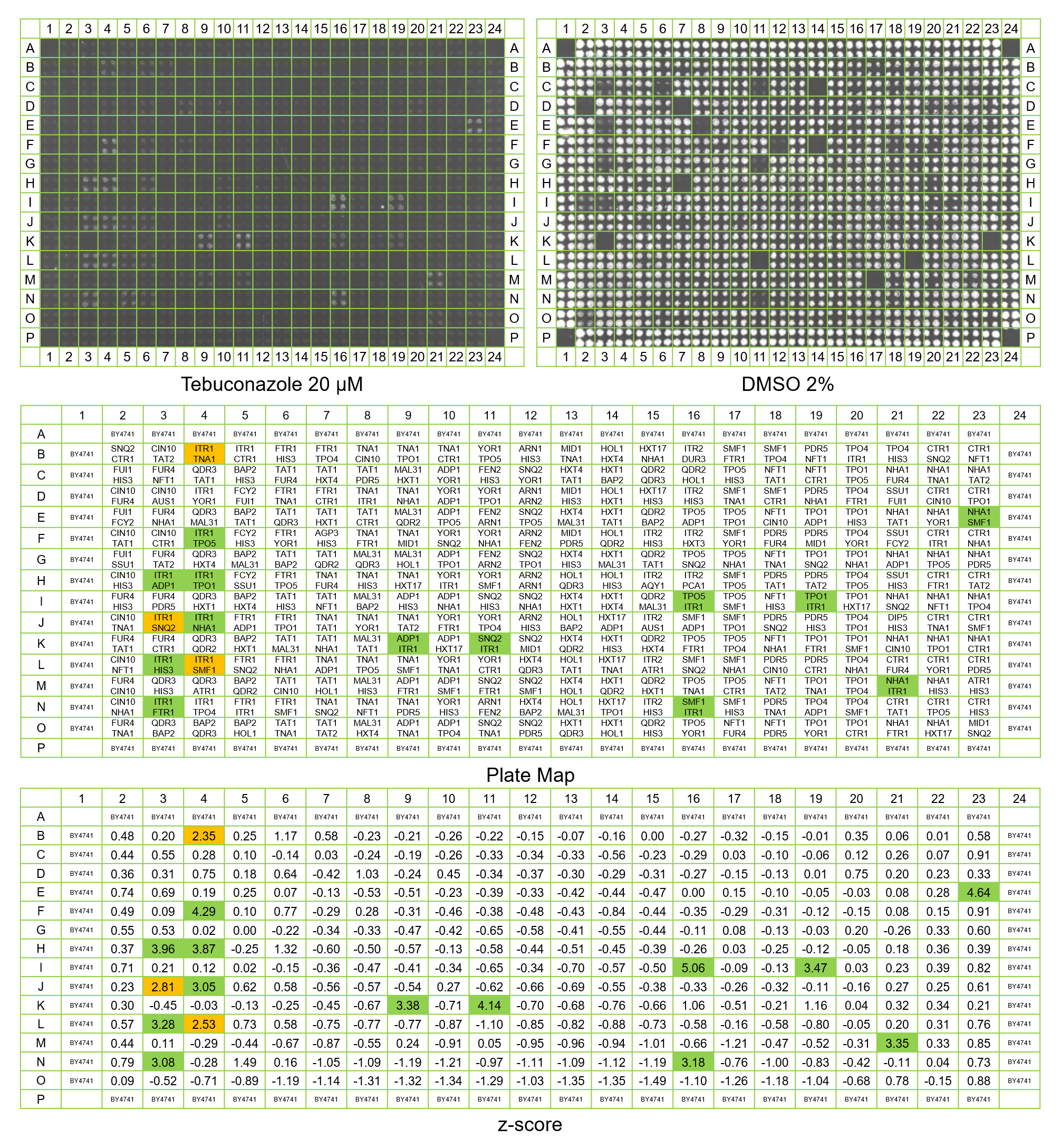

Supplement: FIG S6 [file mbio.03221-21-sf006.tif]

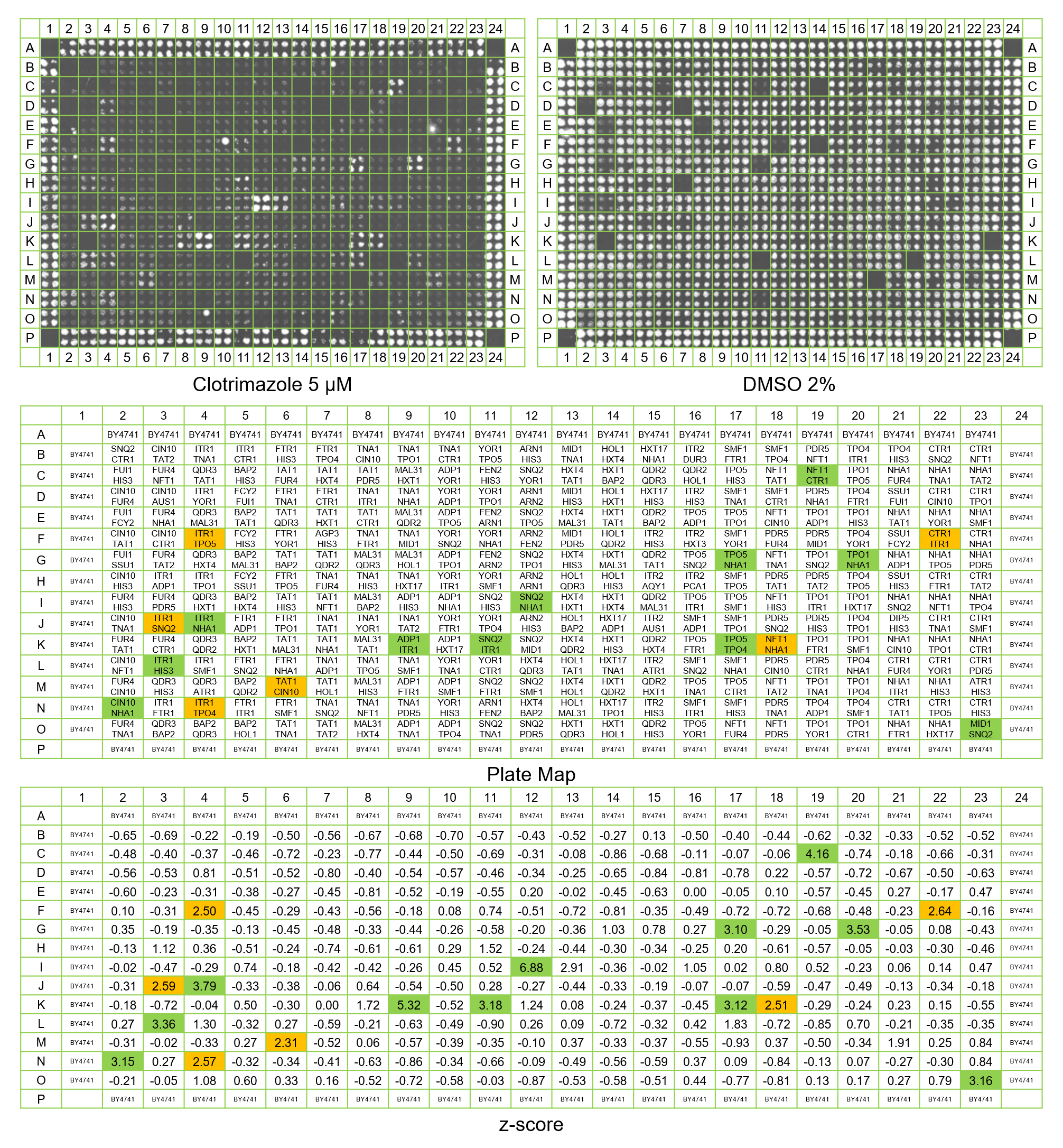

Supplement: FIG S7 [file mbio.03221-21-sf007.tif]

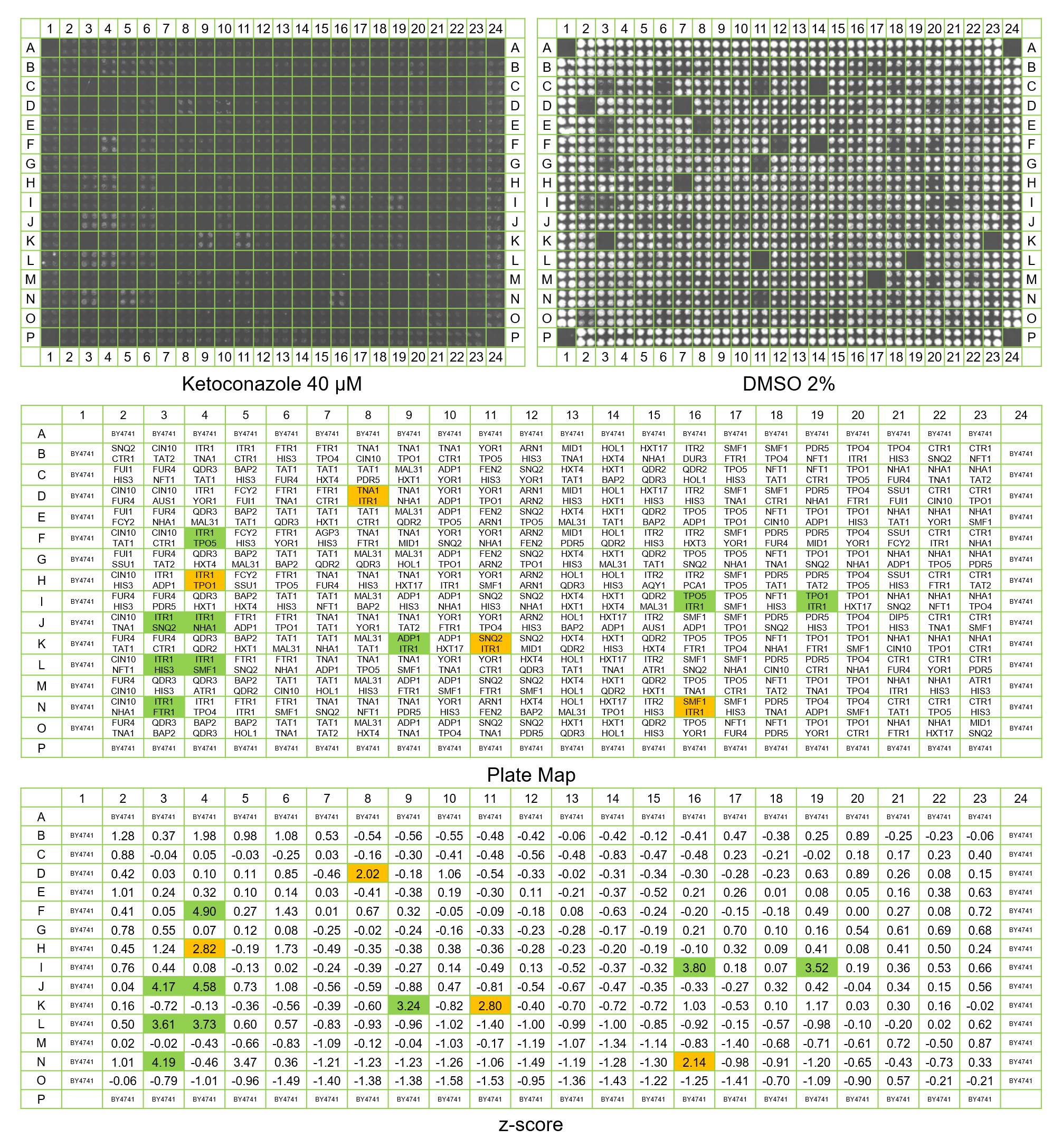

Supplement: FIG S8 [file mbio.03221-21-sf008.tif]
